# Mining and malaria in the Brazilian Amazon and in the Yanomami indigenous land

**DOI:** 10.1371/journal.pntd.0013677

**Published:** 2025-11-03

**Authors:** Marcia C. Castro, Nicholas J. Arisco, Cesar Guerreiro Diniz, Jamie Ponmattam, Cassio Peterka, Paulo Cesar Basta, Marcelo Urbano Ferreira

**Affiliations:** 1 Department of Global Health and Population, Harvard TH Chan School of Public Health, Boston, Massachusetts, United States of America; 2 Environmental Studies Program, Williams College, Williamstown, Massachusetts, United States of America; 3 Solved - Solutions in Geoinformation, Belém, Brazil; 4 Federal University of Pará, Geoscience Institute, Belém, Brazil; 5 Surveillance Department, Amapá State Health Secretariat, Macapá, Brazil; 6 Departamento de Endemias Samuel Pessoa, Fundação Oswaldo Cruz (FIOCRUZ), Rio de Janeiro, Brazil; 7 Department of Parasitology, Institute of Biomedical Sciences, University of São Paulo, São Paulo, Brazil; 8 Global Health and Tropical Medicine (GHTM), Associate Laboratory in Translation and Innovation Towards Global Health (LA-REAL), Institute of Hygiene and Tropical Medicine, NOVA University of Lisbon, Lisbon, Portugal; University of Cincinnati, UNITED STATES OF AMERICA

## Abstract

Illegal mining expanded in the Brazilian Amazon since 2018, leading to increases in malaria among indigenous populations, particularly the Yanomami. We describe the temporal and spatial pattern of malaria and mining in indigenous lands and quantify the impact of mining on malaria among the Yanomami. We estimate that a 1% increase in the annual mining area was associated with a 24% (95% CrI: 17%, 32%) increase in monthly malaria cases in the Yanomami. Also, malaria cases in 2022 in the Yanomami were likely underreported by 83%, and an estimated excess of 102,870 malaria cases occurred from 2018 to 2023 due to increased mining activity (an additional cost to the public health system of approximately US$6.9 million). Rethinking and intensifying malaria control in Brazil is a matter of health, environmental, and indigenous justice.

## Author summary

The expansion of illegal mining in the Brazilian Amazon, and particularly in indigenous lands, has produced severely negative environmental and health externalities. One major health concern is a rising malaria burden among indigenous populations, particularly in the Yanomami indigenous lands, where a humanitarian crisis arose following illegal mining activity. Mining creates highly fragmented forests rife with the ideal habitat for malaria vectors to proliferate. In the Yanomami Indigenous Land, illegal mining grew rapidly from 2018 to 2022 and coincided with a sharp increase in malaria cases. Most malaria cases were linked to zones near mining and along the travel routes used by miners. We found that malaria cases among the Yanomami people are likely greater than official reporting. In 2022, for example, our analysis suggests there were nearly twice as many local malaria cases as the official reports showed. This underreporting may be linked to the destruction of health posts meant to serve indigenous populations. We also found that if mining had not increased beyond 2017 levels, roughly 100,000 fewer malaria cases may have been avoided between 2018 and 2023. These extra cases impose financial burdens on health systems, the Brazilian government, and undue harm to the Yanomami people and their culture. Lasting solutions will require stronger enforcement against illegal mining, better protection of Indigenous peoples’ rights, and close cooperation among sectors like health, law enforcement, and the environment.

## Introduction

In Brazil, more than 99% of malaria cases occur in the Amazon. After more than 600 thousand cases were reported in 2005, malaria began to decline, reaching about 124 thousand cases in 2016, the lowest number in 38 years. In 2017 and 2018, cases increased to almost 190 thousand. After a modest decline after 2018, cases increased again in 2023. What is unprecedented about this recent fluctuation in the number of malaria cases is where the infections are occurring. Brazil’s National Malaria Control and Prevention Program (NMCPP) classifies cases into five types: urban, rural, indigenous, mining, and settlements. Although historically more than half of cases have been reported in rural localities, an increase in cases in indigenous and mining localities has changed this pattern. In 2023, for the first time, malaria cases reported in indigenous localities were the most common, representing over a third of the total cases.

Indeed, in January 2023, a humanitarian crisis was revealed in the Yanomami indigenous land [1,2]. Malnourished children suffering from health complications highlighted the cumulative effects of prolonged exposure to social, economic, and political neglect. The Ministry of Health designated the Yanomami situation a public health emergency of national importance [3]; only twice before has the Ministry of Health issued such an alert: during the Zika epidemic in 2015 and the Covid-19 pandemic in 2020. Three decades earlier, the Yanomami people in Brazil were subjected to a brutal attack that resulted in the deaths of 16 people. This is the only crime recognized as genocide by the Brazilian Supreme Court [4]. The two events that affected the Yanomami, 30 years apart, share a common thread: the expansion of large-scale gold mining (known as *garimpo*), mostly illegal.

During the tenure of former President Bolsonaro, whose political agenda disregarded environmental protection and indigenous rights [5], deforestation and the expansion of illegal gold mining in the Amazon accelerated [6,7]. During the four years of his term (2019–2022), deforestation rates increased by 59.5% compared to the previous four years. Considering all the *garimpo* areas detected from 1985 to 2022, 40% were initiated in just 5 years, from 2018-2002. In indigenous lands this number is 62%. Most importantly, 77% of the *garimpo* showed signs of illegality [8]. The rapid expansion of *garimpo* on indigenous lands is devastating for at least three reasons [9,10]. First, it results in significant environmental damage that extends beyond the area being mined [11,12]. Second, it disrupts indigenous’ traditional food sources, as small crops grown by the indigenous people are taken over by the *garimpo*, and animals that are often hunted as a source of protein run away frightened by the noise of the machinery used in the mining [10]. Third, indigenous health deteriorates: widespread violence, sexually transmitted diseases, malnutrition, diseases related to mercury contamination (a toxic metal used in gold mining), and respiratory infections [9]. In addition, mining activities alter the local environment in ways that promote the proliferation of aquatic habitats suitable for the breeding of *Anopheles* mosquitoes (the malaria vector), thereby contributing to increases in malaria transmission [13].

In light of these facts, the aim of this study is twofold. First, we describe the temporal and spatial pattern of malaria and mining in the Brazilian Amazon, with a special focus on indigenous lands. Second, we specifically quantify the effect of mining on malaria transmission in the Yanomami indigenous land. We use malaria data from the Ministry of Health’s Malaria Epidemiological Surveillance Information System (Sivep-Malaria) and mining data from MapBiomas v.9 (https://brasil.mapbiomas.org/en/).

## Methods

### Yanomami indigenous territory

The Yanomami indigenous land is situated along Brazil’s northern border with Venezuela. It covers an area of approximately 96,650 km^2^, which is comparable to the size of Portugal. The estimated population of the region in 2023 was 31,567. It is inhabited by eight different ethnic groups, some of which have had minimal contact with each other. It is the largest indigenous land in Brazil, extending over two states, Roraima (RR) and Amazonas (AM) in the Brazilian Amazon. A total of 398 indigenous villages (*aldeias* in Portuguese; obtained from: https://infoms.saude.gov.br/extensions/sesai_pop_indigena/sesai_pop_indigena.html#) are distributed across 37 subunits (*polo base* in Portuguese) located in eight Brazilian municipalities, namely Amajari (RR), Alto Alegre (RR), Mucajaí (RR), Iracema (RR), Caracaraí (RR), Barcelos (AM), Santa Isabel do Rio Negro (AM), and São Gabriel da Cachoeira (AM) (Fig A in S1 Text). Of the villages, 301 have been provided with geographical coordinates by the National Indigenous Foundation (Funai; obtained from: https://www.gov.br/funai/pt-br/atuacao/terras-indigenas/geoprocessamento-e-mapas).

### Malaria case data

De-identified malaria cases notified in the Amazon from 2003 to 2023, stored in the Malaria Epidemiological Surveillance Information System (Sivep-Malaria), were obtained from the Ministry of Health. Geographically, every notified case reports the locality, subunit, municipality, and state of infection, residence, and notification. Cases in indigenous lands also report the indigenous subunit. Locality is a sub-national unit used by the NMCPP. At the time of writing, no shapefile of the boundaries of localities was available. Shapefiles for municipalities were obtained from the Brazilian Institute of Geography and Statistics (IBGE), while those for Yanomami subunits were sourced from Natural Earth (Public Domain), https://www.naturalearthdata.com/about/terms-of-use/. We work with two time periods in this analysis: 2003–2023 when considering the entire Brazilian Amazon, and 2010–2023 when specifically analyzing the Yanomami Indigenous Lands. The latter considers a shorter temporal window because population data in each land subunit only extended from 2010 to 2023.

For each malaria case, Sivep-Malaria provides geographic, epidemiologic, demographic, diagnostic, and treatment details. The variables used in this analysis include the municipality of notification and infection; the Yanomami subunit of notification, residence, and infection; the locality of notification and infection; the classification of the infection locality (settlement, urban, indigenous, mining, or rural); the date of symptom onset and notification; and the parasite species. As determined by the NMCPP, individuals who had received malaria treatment within the preceding 40 days (for cases of *Plasmodium falciparum*) or 60 days (for cases of *P. vivax*) were flagged as treatment verification slides and thus excluded from the analysis (n = 15,791) to avoid double-counting cases. The time elapsed between the onset of symptoms and the notification of a case was calculated for both locally acquired and exported cases, with the data stratified by parasite type and the classification of the locality of infection. A total of 36,759 (20.6%) records were missing data on symptom onset. Of those cases without a symptom onset date, 97.8% were locally acquired cases.

Of the cases reported within the states of Roraima and Amazonas that overlap the Yanomami indigenous lands, 6 (0.0005%) cases were missing information on the municipality of infection and 6,899 (0.6%) cases were missing information on locality of infection between 2010 and 2023. The final number of reported malaria cases in the Yanomami indigenous land from 2010 to 2023 included in our analysis was 178,323 (29,925 malaria cases were identified as exported from the Yanomami indigenous land, and 148,398 as locally acquired).

In Brazil, more than 90% of malaria cases are confirmed by blood smear microscopy. Diagnosis and treatment are provided free of charge by the public health system. It should be noted that antimalarial drugs are not available for purchase in pharmacies. However, there have been reports of illegal markets offering these drugs, particularly in mining areas [14].

In accordance with the recommendations of the NMCPP and further informed by the work of Arisco et al. [15], malaria cases were classified as either locally acquired or imported/exported. A locally acquired malaria case is defined as one in which the municipality of infection and the municipality of notification are identical. An imported case is defined as one in which the municipality of infection and the municipality of notification are not identical. This distinction is made possible by the fact that the municipality and locality of infection are assessed at the time of notification, through self-reported travel history in the previous three weeks and the date of symptoms onset.

In the context of the Yanomami indigenous land, a locally acquired case is defined as one in which both the place of infection and the place of notification are within the Yanomami indigenous land; an exported malaria case was defined as one in which the municipality and locality of infection overlapped with a Yanomami subunit different from the municipality and locality of notification. Since the boundaries of the Yanomami subunit do not always align with those of the municipalities, a database of all localities in Brazil (n = 531,261) was used to extract those located within the eight municipalities that overlap with the Yanomami indigenous land (n = 4,138). Of these localities, 515 were located within the boundaries of the Yanomami IL. In addition, 57 localities were not associated with specific Yanomami subunits due to their cross-boundary nature (i.e., *garimpo* localities). However, through a process of literature analysis, an examination of the proximity of mining sites, and a triangulation based on the municipality, we were able to successfully identify the subunit of 27 of these localities. Specifically, any locality that was classified as mining, had the name of a Yanomami subunit in its name, and had evidence of mining activity within the boundaries of that subunit, was assigned to that specific subunit (n = 10). In addition, any locality classified as mining that contained the name of a Yanomami subunit in its name, but had no mining activity within the boundaries of that subunit, was assigned to the neighboring subunit with mining activity that was geographically closest (n = 4). Lastly, a literature search was conducted to determine the location of municipalities within Yanomami subunits (n = 13) [16–20]. We did not include in the analysis 294 cases reported in the 30 localities where the subunit could not be identified. A total of 542 unique localities were located within the boundaries of the Yanomami indigenous land.

### Mining and deforestation data

Considering the established links between malaria and the environmental and social changes driven by mining and deforestation [13,21], we obtained data on these processes. Mining data were extracted from the Brazilian Annual Land Use and Land Cover Mapping Project (MapBiomas – https://mapbiomas.org) Collection 8, using ArcGIS Pro v3.0.0 (ESRI; Redlands, CA). MapBiomas provides data at a resolution of 30 meters per pixel for the entire Brazilian territory, with records dating back to 1985. The mining raster data were converted to polygons, and the mining area (km^2^) within each Yanomami subunit was calculated for the years 2010–2023. Mining sites that had been abandoned and converted into another type of land use were no longer classified as mining by MapBiomas. Thus, the extent of mining operations in a given year may be less than that of the preceding year. Given that the impact of mining extends beyond the immediate mined area [12], buffer zones of 1 km and 5 km were also considered in the vicinity of each mining site. Additionally, data on the location and legal status of airstrips opened on Yanomami indigenous land were obtained from MapBiomas.

The data on deforestation in the Yanomami indigenous land for the years 2010–2022 was obtained from the Brazilian National Institute for Space Research (INPE) [22]. Deforestation has been shown to increase malaria transmission in the Brazilian Amazon, in part by creating vector habitat and increasing human-vector contact [23,24]. However, deforestation in the Yanomami territory occurs for a variety of reasons including mining, indigenous agricultural practices, indigenous housing and communities, and non-indigenous settlement [25,26]. Yanomami communities have used the forest ecosystem sustainably throughout their history [26], highlighting the need to distinguish between deforestation associated with mining and deforestation associated with Yanomami activities. ArcGIS Pro v3.0.0 (ESRI; Redlands, CA) was employed to transform the deforestation raster files into polygons, to deduct the annual extent of mining data obtained from MapBiomas, and to calculate the area of remaining deforestation within each Yanomami subunit for the years 2010–2023.

### Climate data

Monthly aggregated maximum air temperature measured at 2 meters height in Celsius degrees (°C) and total precipitation (cm) data were obtained from the ERA5-Land Global Reanalysis Database for the years 2010–2023 using Python 3 and Google Earth Engine [27]. These data were generated through a combination of meteorological observations and atmospheric models, resulting in a comprehensive global grid of climate data at a resolution of 9 km and with a temporal resolution of 1 hour. The monthly aggregated version of the data, accessible via the Google and Copernicus Climate Data Store, was utilized in this study. As the Yanomami subunits exhibit varying sizes, yet all exceed the 9 km grid size, we calculated the area of each overlapping pixel for each subunit and determined the area-weighted average of the climate variables. Specifically, the area-weighted average maximum 2m air temperature and total precipitation for each month and subunit were calculated by overlaying the ERA5-Land grid (**Fig B in** S1 Text) onto the shapefile of Yanomami territory subunits (**Fig A in** S1 Text). The weighted sum of the climate variable values was calculated by multiplying them by the area of the overlapping pixels and dividing the result by the total area of the subunit.

The Oceanic Niño Index (ONI), which characterizes El Niño or La Niña conditions, was obtained from the National Oceanic and Atmospheric Administration (NOAA) for the years 2010–2023. The ONI is calculated as the three-month moving average of the difference between the sea surface temperatures observed at the present time and the average temperatures recorded between 1980 and 2010. An ONI value exceeding +0.5 denotes the presence of El Niño conditions, whereas a value below -0.5 indicates the occurrence of La Niña conditions. The final month of the three-month moving average period was designated as the exposure month, and the ONI values were incorporated into the dataset in accordance with this designation.

### Descriptive analysis

The spatial distribution of the mean Annual Parasite Index (API), defined as the number of malaria cases per 100 Yanomami people, was mapped by subunit. Mining sites were overlaid with a 5 km buffer to enhance visibility. We evaluated the monthly temporal trajectory of exported and local malaria cases, as well as the cumulative area mined. For each year, a Pearson correlation coefficient was calculated to assess the relationship between malaria cases and the mining area. To illustrate the rapid environmental impact of mining activities, we extracted satellite imagery from the China–Brazil Earth Resources Satellite 4A (CBERS-4) using QGIS Version 3.22 and compared two scenes at two distinct points in time.

### Bayesian spatiotemporal modeling

To quantify the effect of mining on malaria in the Yanomami indigenous land, we used a Bayesian, mixed-effects, zero-inflated negative binomial regression model with spatiotemporal covariance structure. Although monthly cumulative mined areas would more closely approximate relationships in nature, the finest temporal scale of mining data is the year. In addition, the distribution of monthly malaria cases over the study period is right skewed with an overrepresentation of zeros. The outcome variable is monthly malaria cases. Because subunits vary in size, we included mining as the annual cumulative area mined in each Yanomami subunit divided by the area of the subunit. The model had the form:


$$\begin{array}{cc} og\left({MalariaCases}_{jkl}\right)= & \beta_{\text{0}}+\beta_{\text{1}}\left(PercentofSubunitAreaMined_{jl}\right)+\beta_{\text{2}}\left({PercentofSubunitDeforestedwithoutMining}_{jl}\right)\\ & +\beta_{\text{3}}\left({SubunitPopulation}_{jl}\right)+\beta_{\text{4}}\left({AreaofSubunit}_{j}\right)+\beta_{\text{5}}(Month)\\ & +f\left(Temperature_{jk}\right)+f\left(Precipitation_{jk}\right)+f(ONI)+f(Year)+q_{j}+v_{kl}+\varepsilon_{jkl} \end{array}$$

$$q_{j}\sim N\left(\text{0},\sigma_{j}^{\text{2}}\right),u_{j}\sim N\left(\text{0},\sigma_{u}^{\text{2}}\right),v_{kl}\sim N\left(\text{0},\sigma_{kl}^{\text{2}}\right),\varepsilon_{jkl}\sim N\left(\text{0},\sigma \text{2}\right)$$

Where indexes are subunit *j*, month *k*, and year *l*, and the outcome is assumed to follow a zero-inflated Poisson distribution. We included several covariates in the model (Table A in S1 Text) to minimize potential confounding of the relationship between annual cumulative area mined, defined as a percentage of the subunit area $\beta_{\text{1}}\left(PercentofSubunitAreaMined_{jl}\right)$, and monthly malaria cases (${MalariaCases}_{jkl}$). The covariates included $\beta_{\text{2}}\left({PercentofSubunitDeforestedwithoutMining}_{jl}\right)$, the proportion of the subunit area that was deforested (not related to mining); $\beta_{\text{3}}\left({SubunitPopulation}_{jl}\right)$, the estimated annual population of each subunit in each year; $\beta_{\text{4}}\left(A{reaofSubunit}_{j}\right)$, the area of each subunit; and $\beta_{\text{5}}(Month)$, a fixed effect for month of the year. Because weather and climate patterns influence malaria, we included nonlinear random walk terms for ONI (2^nd^ order, f(ONI)) and monthly total precipitation (1^st^ order, $f\left(Precipitation_{jk}\right)$), as well as autoregressive terms for monthly maximum temperature (2^nd^ order, $f\left(Temperature_{jk}\right)$), to approximate fluctuations in seasonal weather patterns and potential extreme events (Fig C in S1 Text). To control for spatiotemporal dependencies in the data, we included random intercepts for months nested within year ($v_{kl}$) and subunit ($q_{j}$), a 1^st^ order autoregressive term for year (f(Year)), a fixed effect for month to control for within-year variability ($\beta_{\text{5}}(Month)$), and spatial correlation structure for subunits specified via a Besag-York-Mollier model ($u_{j}$) [28]. $\varepsilon_{jkl}$ is the individual observation error term.

We employ conservative informative priors for both the regression parameters and the hyperparameters (Table B in S1 Text). We ran the spatiotemporal Bayesian model for different temporal stratifications to assess the variability of the model coefficients over time. Three distinct temporal periods were considered: January 2010 to December 2023 (the entire time series), January 2010 to December 2017, and January 2018 to December 2023. Additionally, distinct models were constructed to examine the outcomes of total malaria cases and locally acquired cases exclusively. The full posterior distribution was estimated based on 10,000 samples from each of the six time-stratified models.

The posterior marginal distributions were estimated using Integrated Nested Laplace Approximations (INLA), with the objective of optimizing computational efficiency [29,30]. The mean number of cases for each posterior distribution is reported, along with the respective 95% credible intervals. All models were executed in R (R Core Team, 2020), utilizing the ‘ggplot2’, ‘INLA’ (www.r-inla.org), and ‘spdep’ libraries [31,32].

To ensure the selection of a robust model, we conducted a series of sensitivity analyses. First, we tested different specifications to control for the effects of time and season. All possible combinations of fixed effects and random effects terms for year and month were assessed, as well as a smoothed cosine term to emulate monthly malaria seasonality. Once the variable selection process was complete, the suitability of different distributional assumptions for the model outcome was tested, including Poisson, negative binomial, zero-inflated Poisson, and zero-inflated negative binomial distributions. The model’s predictive power was evaluated through leave-one-out cross-validation, which entailed comparing the cumulative conditional predictive ordinates (CPO) for each observed data point. Additionally, the deviance information criterion (DIC), marginal likelihood, and the Watanabe–Akaike information criterion (WAIC) metrics were employed to compare performance across models.

### Simulated scenario

Given that locally acquired malaria cases in the Yanomami indigenous land declined in 2022, despite the significant increase in mining activity, we estimated the likely number of locally acquired cases that may have occurred in 2022. To that end, we fit the previously described spatiotemporal Bayesian model to the entire time-period, 2010–2023, with the outcome values for 2022 set to null and all other variables held at their observed levels. We calculated the monthly median number of locally acquired malaria cases from the posterior distribution and the corresponding 95% credible intervals, as well as the difference between observed and predicted cases for 2022.

We simulated a counterfactual scenario in which the area mined from 2018 to 2023 did not increase exponentially, but rather mimicked historical patterns observed from 2010 to 2017. We calculated the mean area mined within each Yanomami subunit from 2010 to 2017 and created a distribution of the mean and one-tenth of the standard deviation of the area mined during that period for each subunit as a measure of random variability. For each subunit and year between 2018 and 2023, we added a randomly selected value from the distribution mimicking random variability to the mean area mined from 2010 to 2017. All other covariates in the model were kept at their observed levels. To estimate the malaria cases that would have been associated with the counterfactual mining levels, we fit the same previously described spatiotemporal Bayesian model to the dataset including all years, 2010–2023, with the outcome from 2018 to 2023 set to null. We calculated the monthly median number of cases from the posterior distribution and the respective 95% credible intervals. We also calculated the difference between observed and predicted malaria cases for the years 2018–2023.

## Results

### Recent trends in malaria and mining in the Amazon

**Fig 1:**
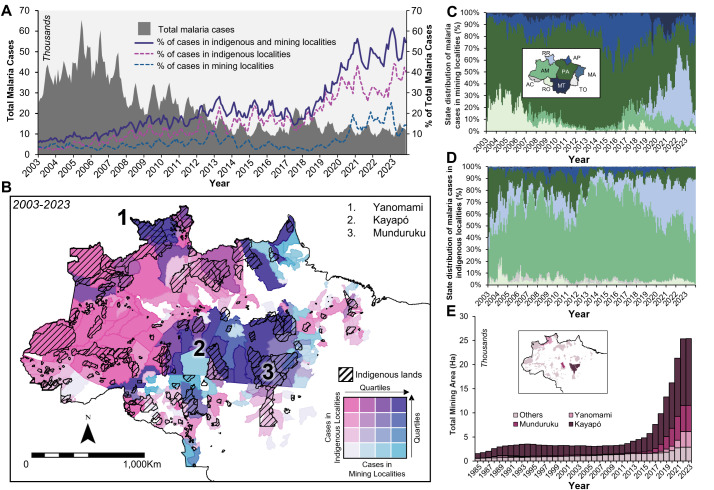
Malaria cases and mining in indigenous areas of the Brazilian Amazon. (A) Monthly malaria cases in the Brazilian Amazon, and percentage of cases in mining and indigenous localities, 2003-2022. (B) Bivariate map of cumulative malaria cases in indigenous and mining localities from 2003 to 2023. Hashed areas correspond to demarcated indigenous lands. Yanomami, Kayapó, and Munduruku indigenous lands (numbered on the map) are the most affected by illegal mining. (C) Percentage of total monthly malaria cases in mining localities by state. State acronyms AC = Acre, AP = Amapá, AM = Amazonas, MA = Maranhão, PA = Pará, RO = Rondônia, RR = Roraima, and TO=Tocantins. The color of the area in the graph corresponds to the color of the state in the map. (D) Percentage of total monthly malaria cases in indigenous localities by state. The color of the area in the graph corresponds to the color of the state in the map shown in C. (E) Cumulative area converted to mining in indigenous lands, 1985 to 2023. Yanomami, Kayapó, and Munduruku indigenous lands are highlighted. Basemap accessed at Natural Earth (Public Domain), https://www.naturalearthdata.com/about/terms-of-use/.

A time series of daily malaria cases from 2003 to 2023 shows a decline over time, while the percentage of cases originating in indigenous and mining localities shows the opposite trend (**Fig 1A**). Between 2016 and 2023, annual cases increased in indigenous localities from 21,628–54,796 (153.4% increase) and in mining localities from 5,500–20,288 (268.9% increase), respectively. Taken together, cases in these localities accounted for only 6.8% of all malaria cases in the Amazon in 2003 but increased to 52.8% in 2023. These cases largely overlap with areas demarcated as protected indigenous lands (**Fig 1B**).

The relative distribution of malaria cases in mining localities has shifted among states in the Amazon. In 2003, 72.2% (11,947 cases) of malaria cases in mining localities were concentrated in Pará and only 0.2% (26 cases) in Roraima. These Figs changed to 42.9% (8,696 cases) and 31.9% (6,483 cases), respectively, in 2023 (**Fig 1C**). Regarding cases reported in indigenous localities, Amazonas state consistently concentrated the larger share (38.8%, 4,423 cases in 2003, and 43.9%, 24,059 cases in 2023), and Roraima more than tripled its share between 2003 (13.2%, 1,502 cases) and 2023 (42.8%, 23,431 cases) (**Fig 1D**). The Yanomami indigenous land extends over these two states.

Considering mining activities in the Amazon from 1985 to 2022, the expansion of illicit gold mining on indigenous lands was particularly pronounced in three areas: Kayapó and Munduruku in Pará, and Yanomami in Roraima (**Fig 1E**). Between 2012 and 2022, the area mined increased by 622.0% (19.1 km^2^ to 137.8 km^2^) in the Kayapó, 2,224.7.2% (2.35 km^2^ to 54.6 km^2^) in the Munduruku, and 73,274.0% (0.05 km^2^ to 36.7 km^2^) in the Yanomami. In 2023, the first year of Presidente Lula’s term, the opening of new illegal mining areas stalled.

### Mining expansion and malaria in the Yanomami indigenous land

**Fig 2:**
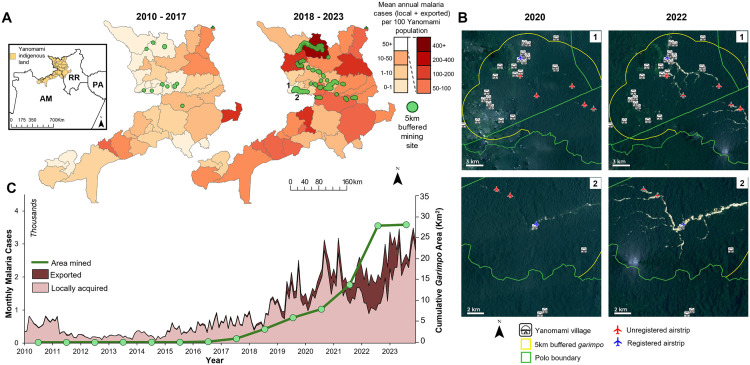
Malaria cases and mining in the Yanomami indigenous land. **(A)** Mean annual malaria cases per 100 people in Yanomami villages between 2010 and 2017 and 2018 and 2023. Green areas represent a 5km buffer zone around *garimpo*. The inset map shows the location of the Yanomami territory, which spans two states, namely Roraima (RR) and Amazonas (AM). **(B)** Satellite images comparing pre- (2020) and post-*garimpo* (2022) activity in two areas of the Yanomami indigenous land, numbered as 1 and 2 in the 2018-2023 choropleth map in **A**. The white area in 2022 shows the environmental change following the mining activity along the river. The high-resolution images are courtesy of Norway’s International Climate and Forest Initiative (NICFI Program), acquired through its official Planet-NICFI mosaics API. The pair of images are monthly mosaics from September 2020 and September 2022; **(C)** Cumulative area mined and monthly malaria cases in the Yanomami indigenous land between 2010 and 2023. Cases are disaggregated by exported and locally acquired. Basemap accessed at Natural Earth (Public Domain), https://www.naturalearthdata.com/about/terms-of-use/.

In 2010, mining activities covered 0.03 km^2^ of the Yanomami indigenous land. By 2022 this area had increased to 36.7 km^2^, and did not change in 2023 (Table C in S1 Text). Mining expansion was intense from 2018 to 2022. Of the 37 subunits in the Yanomami indigenous land, five – Waikás, Paapiu, Homoxi, Auaris, and Xitei (Fig 2A and Fig A in S1 Text) – concentrated 87.4% of all mining in 2023. The subunit Xitei (Fig 2B, scene 1) had no mining activity in 2020 but showed a substantial increase in 2022, and Homoxi (Fig 2B, scene 2) increased from 0.2 km^2^ in 2020 to 6.1 km^2^ in 2023. The average distance from the villages to *garimpo* areas in Paapiu and Waikás was 0.25 km and 1.1 km, respectively. There were 75 airstrips in the Yanomami indigenous land, 56 of which were unregistered and therefore illegal. Thirteen of the 37 subunits had an average distance between villages and airstrips smaller than 5 km. The minimum distance was observed in Auaris (59 m), and 20% of the villages were less than 1 km from an airstrip.

The number of malaria cases, as recorded in Sivep-Malaria, also increased in the Yanomami indigenous land (Table D in S1 Text). Between 2010 and 2017, when the cumulative *garimpo* area was 1.23km^2^, malaria increased at an average annual growth of 9.5%, while between 2018 and 2023, following the increase in the area mined, malaria increased at an average annual growth of 30.2%. We calculated the annual average of malaria cases per 100 people by subunit for the years 2010–2017, and for 2018–2023 (**Fig 2A**). Although we observe an increase in the latter period, following the rapid expansion of mining, the number of cases in 2022 (n = 25,650) was slightly smaller than 2021 (n = 25,682). In 2023, however, when mining activity was the same as 2022, 35,506 cases were reported (**Fig 2C**), suggesting underreporting of cases in 2022. In addition, we observe high Annual Parasite Indices (API, number of malaria cases per 100 people) in subunits without any mining activity, such as Uraricoera and Palimiú (Table D in S1 Text). It is important to note that one of the routes used by the miners to bring supplies and equipment from Roraima’s state capital to the mining sites passes through these subunits.

**Fig 3:**
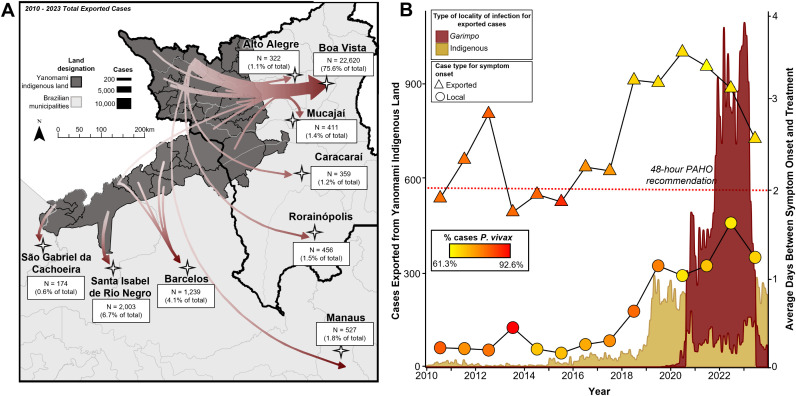
Malaria cases exported from the Yanomami indigenous land. **(A)** Arrows indicate the direction of 93.9% (28,111 of 29,925) of the destinations of cases exported from the Yanomami territory between 2010 and 2023. **(B)** Monthly number of malaria cases exported from the Yanomami territory whose locality of infection was mining or indigenous. The lines indicate the average number of days between the onset of malaria symptoms and treatment for locally acquired (represented by a circle) and exported (represented by a triangle) cases. The shades of the circles and triangles represent the percentage of *P. vivax* cases. The dashed red line indicates the maximum time from symptom onset to treatment recommended by the Pan American Health Organization (PAHO). Does not include 36,759 (20.6%) records missing data on symptom onset. Light grey basemap accessed at: geoBoundaries (CC BY 4.0). Source: https://www.geoboundaries.org/countryDownloads.html. Basemap accessed at Natural Earth (Public Domain), https://www.naturalearthdata.com/about/terms-of-use/.

To account for the mobility of individuals infected with malaria, we classified malaria cases as locally acquired and exported. We observe that the decline in malaria in 2022 was mainly in locally acquired cases, while exported cases continued to increase (**Fig 2C**). This is intriguing and contradicts reports of a worsening malaria burden among the Yanomami people [2,9,33]. To further investigate this decline, we assessed the routes of exported cases, and the time it took between the onset of malaria symptoms and treatment. Between 2010 and 2023, 29,925 malaria cases were exported from the Yanomami indigenous land, 93.4% of which occurred between 2019 and 2023, with a peak of 10,617 in 2022. The most common origin within the Yanomami indigenous land was the subunit of Waikás (20,062 cases exported between 2010 and 2023), the subunit with the largest mining area (12,7 km^2^) (Table C in S1 Text). The most common destination of exported cases was Boa Vista, the capital of the state of Roraima, and the largest city in the vicinity of the Yanomami indigenous land (**Fig 3A**). Of all malaria cases imported to Boa Vista in 2023 (n = 7,182), 4,111 (57.2%) originated from the Yanomami indigenous land, and in 70.4% of these cases (n = 2,896) the infected person reported mining as an economic activity. Until September 2019, all malaria cases exported from the Yanomami indigenous land originated in indigenous localities (**Fig 3B**). Afterwards this pattern changed. The number of cases exported from mining localities changed from 1 in September 2019–978 in December 2023. The total number of cases exported from the Yanomami indigenous land peaked in March 2022 at 1,040 cases, of which 94% were from mining localities.

In terms of time from malaria symptoms onset to treatment, cases exported from the Yanomami indigenous land had a longer average time to treatment than locally acquired cases, mostly beyond the 48-hour window recommended by the Pan-American Health Organization (**Fig 3B**). The distribution of malaria parasite species of reported cases also changed. While in 2010 14.9% and 84.5% of locally acquired cases were diagnosed as *Plasmodium falciparum* and *P. vivax*, respectively, in 2023 these Figs changed to 26.6% and 67.2%, respectively. For exported malaria cases, *P. falciparum* cases changed from 9.7% in 2010 to 23.3% in 2023, and *P. vivax* from 87.0% to 69.2% over the same period.

It is critical to note that gametocytes (the parasite stage ingested by female mosquitoes after biting an infected person) usually appear before the onset of symptoms in *P. vivax* cases but take between 7 and 10 days to appear after the onset of symptoms in *P. falciparum* cases. Despite the recent increase in *P. falciparum* cases, our results highlight two issues. First, the increase in both exported cases and those acquired in mining localities suggests the persistent presence of a local reservoir of parasites, often enhanced by the delay in treatment. Therefore, it is epidemiologically unreasonable that locally acquired cases would decline, while exported cases continued to increase. Second, as exported cases leave the Yanomami indigenous land, *P. vivax* infections (about two-thirds of exported cases) potentially infect mosquitoes in areas along the route to destination. This supports the increase in malaria in subunits where there is no mining activity, as highlighted above (**Fig 2A**).

### The impact of mining on malaria

**Fig 4:**
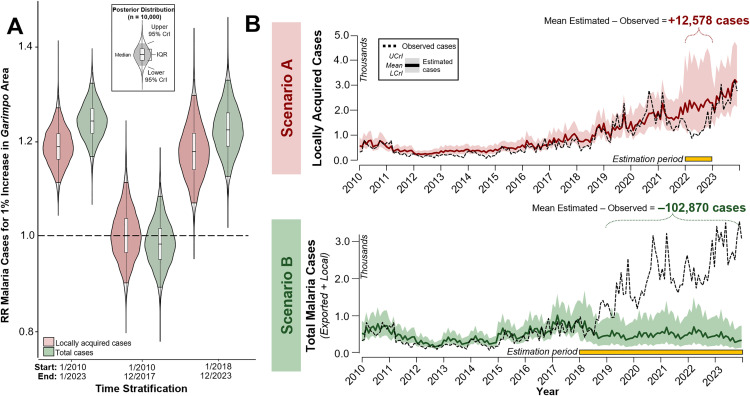
Models of malaria and mining in the Yanomami indigenous land. (A) Posterior distribution based on 10,000 samples for time-stratified spatiotemporal Bayesian models considering monthly malaria cases from Jan/2010 to Dec/2023, Jan/2010-Dec/2017, and Jan/2018-Dec/2023 for both locally acquired cases and total malaria cases as outcomes. (B) Monthly malaria case estimates considering two simulated scenarios. Scenario A estimates the number of monthly malaria cases during the period of suspected underreporting (January/2022 to Dec/2022). Scenario B assumes that mining activity between 2018 and 2022 mimics the pattern observed before 2018 and estimates the number of monthly cases from Jan/2018 to Dec/2023. In both scenarios, all other covariates enter the model as observed. UCrI and LCrI indicate the upper and lower, respectively, 95% credible interval.

We used a spatiotemporal Bayesian framework to fit zero-inflated negative binomial models. The outcome was the number of monthly malaria cases that originated in the Yanomami indigenous land (Jan/2010 to Dec/2023). The primary exposure is the proportion of the subunit area with mining activity. A 1% increase in the annual area mined was associated with a 24% (95% CrI: 17%, 32%) increase in monthly malaria cases in the Yanomami indigenous land between Jan/2010 and Dec/2023 (Table F in S1 Text). Given the decline in locally acquired malaria cases, a model was constructed in which this variable was the outcome; the impact of mining was less pronounced (19%; 95% CrI: 11%, 27%). Furthermore, due to the exponential increase in mining after 2017, separate models were constructed for the periods Jan/2010-Dec/2017 and Jan/2018-Dec/2023. In the first model, the effect of mining on malaria was not statistically significant. In the second model, a 1% increase in the annual area mined was associated with a 22% (95% CrI: 13%, 33%) increase in monthly malaria cases (Fig 4A and Tables F and G in S1 Text). Because mining causes deforestation beyond the area mined [12], we also ran models that consider the percentage of mining in each subunit, as described by a 1km and a 5km buffer around the mining site. Some mining sites are close to each other, therefore overlapping buffer areas were not counted twice. Overall, the effect of mining on total malaria cases was similar at 1km buffer but was larger at 5km (34%; 95% CrI: 25%, 44%).

Sensitivity analyses were conducted on the model and time specifications (Tables H and I in S1 Text). We tested twelve different time specifications, including random, non-linear, and fixed effects for month and year, and compared models using the DIC, WAIC, and the log of the conditional predictive ordinate (a leave-one-out cross validation tool for INLA models). We used these same metrics to compare model fit based on four distributional assumptions: zero-inflated Poisson, zero-inflated negative binomial, negative binomial, and Poisson.

### Simulated scenarios

Using the same spatiotemporal Bayesian framework, we estimated the number of locally acquired malaria cases in 2022, given the observed mining activity. Instead of the 15,033 locally acquired cases reported in 2022, we estimated a total of 27,611 cases, or almost twice the number reported (Fig 4B, Scenario A and Table J in S1 Text). The largest relative underreporting was estimated to occur in the Homoxi (338 cases against only 1 reported), Haxiu (46.4 times the number of reported cases), Xitei (33.6 times the number of reported cases), and Ajarani (22.7 times the number of reported cases). According to reports from the Ministry of Health, the health units in these subunits were not operational in 2022 [34]. In fact, Homoxi and Haxiu reported malaria cases in only one month of 2022, while Xitei and Ajarani reported cases in only 6 and 4 months, respectively.

In addition, we examined a scenario in which the exponential increase in mining on the Yanomami indigenous land after 2017 did not occur. We estimated that 36,366 malaria cases would have occurred between 2018 and 2023 (instead of the 139,236 cases reported) had mining activities mirrored the pattern prior to 2018 (Fig 4B, Scenario B, and Table J in S1 Text). This represents an estimated excess of 102,870 malaria cases from 2018 to 2023 due to increased mining activity, which is 2.6 times the number of cases reported from 2010 to 2017. The largest excess of malaria cases between 2018 and 2023 was estimated to occur in the Waikás subunit (21,441 cases), which concentrates 35% of the total mining area in the Yanomami indigenous land (Table A in S1 Text).

## Discussion

In this study, we examined the evolution of malaria cases in the Brazilian Amazon, focusing on those reported in indigenous communities. We also evaluated the recent surge in mining activity on Yanomami indigenous land and quantified its impact on reported malaria cases. After controlling for weather, our results show that a 1% increase in the annual area mined was associated with a 24% (95% CrI: 17%, 32%) increase in monthly malaria cases in the Yanomami indigenous territory between January 2010 and December 2023. Furthermore, we demonstrate that malaria cases in 2022 were likely underreported due to the closure of several health units caused by the insecurity imposed by mining activities. Additionally, we estimate that more than 100 thousand malaria cases between 2018 and 2023 would not have occurred if mining had not increased sharply in the Yanomami indigenous territory.

The impact of illegal mining on malaria, particularly among indigenous populations, is one of the many effects of social, economic, and political neglect. A tool developed to measure the social and environmental costs of illegal gold mining in the Amazon [35] estimates that these costs are more than ten times the profits made by those who trade in gold. Since the late 1980s, when gold was found in the Yanomami indigenous land, illegal mining has contributed to epidemics of respiratory infections and malaria. Between 1987 and 1990, approximately 14% of the Yanomami living in Roraima died as a result of mining activities [36]. The use of mercury in *garimpo* has resulted in the contamination of rivers, fish, plants, and the air, leading to a range of adverse health effects [37]. A recent study in the Alto Mucajaí subunit, found mercury contamination in all individuals and fish analyzed, cognitive deficiencies, and severe malnutrition, among other problems [38–40].

Increases in malaria associated with mining have been reported in other countries that share portions of the Amazon, as well as in countries in Southeast Asia and Africa [13,41–43]. This is particularly concerning in areas close to indigenous populations [44–47]. Here we show that the unprecedentedly rapid expansion of illegal mining in the Yanomami indigenous land in Brazil was accompanied by a significant increase in malaria. With the declaration of a public health emergency of national importance in Jan/2023 [48], several missions to the area revealed the precarious situation. In seven subunits (Paapiu, Homoxi, Hakoma, Ajaraní, Haxiú, Xitei, and Palimiú), health units were not functioning due to lack of security. In some of them, the buildings were destroyed, health professionals had to leave the area, and equipment and medicines were stolen by miners [49]. This left more than 5,200 Yanomamis without access to health care. These units came back into operation in 2024 [34]. These results raise two critical discussions.

First, although the link between mining and malaria is well established [13,41,50], the crisis observed in the Yanomami indigenous land was amplified by political decisions and thus could have been avoided. Under President Bolsonaro (2019–2022), environmental policies were weakened, and indigenous rights were threatened, all part of an agenda that favored market interests and agribusiness, ignoring social, health, environmental, and cultural impacts [49,51–53]. In addition, court rulings were overlooked, such as the need to implement safety and health measures [49]. Moreover, the government largely ignored multiple requests from various stakeholders warning of the unfolding health crisis in the area. Just one civil society organization sent more than 37 requests between 2021 and 2022 [49].

Second, addressing the impact of illegal mining on malaria requires intersectoral collaboration that brings together health, environment, national security, the armed forces, mining and energy, and human rights. For example, the involvement of criminal factions in *garimpo* came to light in 2021, when criminals attacked communities in the Palimiú subunit, openly shooting women and children [49]. Many other incidents of violence against indigenous people and health workers make it clear that no health operation can be carried out safely without security support. In addition, it is imperative to restore and enforce environmental regulations to curb illegal mining, to establish appropriate mechanisms to regulate the market, and to develop innovative local business models that discourage illegal mining. Intersectoral and collaborative efforts that include traditionally marginalized voices – such as women, youth, local communities, and indigenous peoples – are the basis for sustainable development [54]. However, development models implemented in the Amazon have been historically based on resource exploitation, ignoring local well-being and needs [55].

With emergency measures implemented throughout 2023, no further illegal mining was reported in the Yanomami indigenous land that year. The operations have disabled machines, boats, aircrafts, engines, fuel, weapons, and antennas. But the problem is far from over. The number of malaria cases in the Yanomami indigenous land reached 35,506 in 2023 and 33,463 in 2024 (preliminary data). In 2024, 42,3% of all malaria cases reported in the Brazil occurred in indigenous localities, and the Yanomami respond for almost 60% of all malaria cases in indigenous localities.

In addition, the number of reported malaria cases is only a fraction of the number of malaria infections in the area. In the Brazilian Amazon, most malaria cases are diagnosed by microscopy (above 90% until 2019, and around 86% between 2020 and 2023). In the Yanomami indigenous land, the use of antigen-based rapid diagnostic tests (RDTs) has been expanding since 2018, although most cases are still diagnosed by microcopy (in 2023, 26% of the cases in the Yanomami indigenous land were diagnosed by RDT, and 74% by microscopy) (Fig D in S1 Text). However, microscopy and RDTs may have low sensitivity for malaria diagnosis [56]. During a rapid epidemiological assessment conducted in nine villages of the Alto Mucajaí subunit in October 2022, we found that only 9.4% (3 of 32) of the infections diagnosed by quantitative PCR targeting mitochondrial DNA sequences of malaria parasites had been detected by field microscopy and RDTs [38]. Specifically, of the 273 individuals tested, 32 (11.7%) were positive for malaria parasite DNA. Of these, 24 had *P. vivax* (including the 3 participants who were positive by field microscopy and RDTs), seven had *P. falciparum*, and one had a mixed infection with both parasites. All infections missed by microscopy and RDTs were asymptomatic.

In addition to diagnostic challenges, the percentage of malaria cases in the Brazilian Amazon that are passively detected has remained roughly stable around 75% (Fig E in S1 Text). In the Yanomami indigenous land, more than 70% of cases were actively detected until 2017. However, from 2018, when mining also began to expand in the area, active detection decreased, reaching its lowest level in 2022 (only 36.4% of malaria cases were actively detected), the year in which we estimate a likely underreporting of around 83%. Given the security threats and destruction of health facilities associated with illegal mining, the decline in active case detection and the reliance on microscopy in the Yanomami indigenous land suggests a large underreporting of the true burden of malaria infection.

Our results, and the recent increase in malaria in 2023 and 2024, highlight the need to rethink and intensify control efforts towards elimination. In 2022, Brazil launched an elimination plan with the goal of achieving zero malaria cases and deaths by 2035 [57]. We argue that without a multisectoral approach [58] and the use of surveillance as an intervention (a pillar of the Global Technical Strategy for Malaria [59]), the elimination goal will be difficult to achieve. Here, surveillance also includes specific protocols for active case detection in indigenous lands and for the use of loop-mediated isothermal amplification (LAMP) assays to detect asymptomatic infections [60]. Multisectoral efforts can (and should) be informed by the diverse data available in Brazil. These include malaria surveillance, land use change alerts, airstrip inventory, land conflicts, weather, etc.

This study has limitations. Data on mining area were only available by year, which does not allow modeling the seasonality of malaria and mining, nor does it allow for biologically relevant or informative lag structures to be incorporated into model frameworks. In addition, malaria case data are subject to reporting error for several reasons. First, disruptions in care (e.g., closure of health facilities) affect surveillance and case reporting (as critically observed in 2022). Second, administrative records that leverage self-reporting are subject to bias and incorrect reporting. Some miners and other individuals may not properly report their travel history, so there may be some underreporting of malaria cases, both exported and local. Third, it is possible that some malaria cases among miners were self-treated and thus never reported; this hypothesis is based on reports of drugs stolen by miners [10]. Fourth, since the data used in the study are de-identified, there is no way of formally testing for re-infection of individuals. However, re-infection is likely occurring given the size of the Yanomami population and the total malaria cases reported in each year. Lastly, asymptomatic infections are often not detected by surveillance systems that mostly rely on passive case detection. Therefore, our results should be considered as a conservative estimate of the impact of mining on malaria.

Although this study focused on malaria, it is important to emphasize that the health impacts of mining are much broader, with long-term consequences such as impaired child development. Sexual violence, girls and women forced into prostitution, people co-opted into mining, alcoholism, mercury contamination, and the disruption of traditional norms and values are among the many problems faced by the Yanomami and other indigenous peoples threatened by illegal mining [49,61–64]. In addition, during the Covid-19 pandemic, the spread of SARS-CoV-2 was also linked to mining and miners mobility [65]. It is important to note that, during the Covid-19 pandemic, the number of malaria cases reported in the country was slightly lower than those reported in 2019 (156,916 in 2019, 123,387 in 2020, 139,943 in 2021, and 130,710 in 2022) [66], despite initial expectations that a surge in cases could occur due to disruptions in care observed during the pandemic [67]. Malaria diagnosis and treatment in the Amazon are delivered through a dedicated network of community posts that notify cases to SIVEP-Malaria, and continuity of these services was explicitly prioritized during COVID-19 [68,69]. Nevertheless, although the number of malaria cases did not increase during the pandemic (from 2020 to 2022), compared to 2019, mining and indigenous localities did experience an increase in malaria cases. This underscores the need to address illegal mining and its far-reaching consequences, ensuring the protection of indigenous communities, their health, and their culture.

## Supporting information

S1 Text Fig A in S1 Text. Yanomami subunits and villages. The green dots are the location of 301 villages that were mapped by the National Indigenous Foundation (Funai). The Indigenous Health Secretariat (SESAI) reports a total of 398 villages. Therefore, 97 villages do not have geographical coordinates. The numbers in the maps are the ID# of each subunit (*polo base*), as shown in the table below. Basemap: Natural Earth (Public Domain) https://www.naturalearthdata.com/about/terms-of-use/. Administrative boundaries (Brazil): geoBoundaries (CC BY 4.0) https://www.geoboundaries.org/countryDownloads.html. Indigenous lands (Brazil): FUNAI – Terras Indígenas (open government data; attribution required - see FUNAI page for terms) Main portal: https://www.gov.br/funai/pt-br/atuacao/terras-indigenas/geoprocessamento-e-mapas, Dataaccess/preview: https://geoserver.funai.gov.br/geoserver/web/wicket/bookmarkable/org.geoserver.web.demo.MapPreviewPage?filter=false. **Fig B in S1 Text. Grid of weather data extracted from ERA5.** Weather variables (temperature and rainfall) were aggregated to their associated subunit (*polo base*) by taking the weighted average of the area of the cells that overlapped each subunit. Basemap: Natural Earth (Public Domain) https://www.naturalearthdata.com/about/terms-of-use/. **Fig C in S1 Text. Partial autocorrelation function (PAF) of climate variables by month**. **(A)** Maximum temperature. **(B)** Total precipitation. **(C)** Oceanic Niño Index. **Fig D in S1 Text. Diagnostic method used in reported malaria cases in the Brazilian Amazon and in the Yanomami indigenous land, 2014–2023.** There was no missing information on diagnostic method except for the entire Amazon (1.8% in 2014, only 0.1% in 2023). **Fig E in S1 Text. Type of detection (passive or active) of reported malaria cases in the Brazilian Amazon and in the Yanomami indigenous land, 2014–2023.** There was no missing information on the type of detection. **Table A in S1 Text. Variables included in the models**. Two models were considered: (i) all malaria cases, and (ii) only locally acquired malaria cases. **Table B in S1 Text. Prior specifications across all INLA models**. Prior specifications were chosen to be conservative in their information. **Table C in S1 Text. Area of *garimpo* in the Yanomami indigenous land by subunit, 2010–2023**. ID number corresponds to the map in Fig A in S1 Text. **Table D in S1 Text. Reported malaria cases in the Yanomami indigenous land by subunit of infection, 2010–2023**. ID number corresponds to the map in Fig A in S1 Text. **Table E in S1 Text. Malaria Annual Parasite Index (API) per 100 people in the Yanomami indigenous land by subunit of infection, 2010–2023**. ID number corresponds to the map in Fig A in S1 Text. **Table F in S1 Text. Model results for different time periods, outcomes, and configuration of mining data. Table G in S1 Text. Additional fixed effects included in model (relevant to model that used observed mining as the outcome). Table H in S1 Text. Sensitivity analysis of the temporal specification of the model**. For DIC and WAIC, lower numbers indicate a better fit; for logCPO, higher numbers indicate a better fit. The model specification used for both locally acquired malaria cases and total malaria cases is indicated by an asterisk (*). **Table I in S1 Text. Distributional assumption sensitivity analysis**. For DIC and WAIC, lower numbers indicate a better fit; for logCPO, higher numbers indicate a better fit. The model specification used for both locally acquired malaria cases and total malaria cases is indicated by an asterisk (*). **Table J in S1 Text. Estimates for simulated scenarios.** Scenario A = estimated number of locally acquired malaria cases in 2023. The difference between estimated and reported cases refers to the likely underreporting of locally acquired cases. Scenario B = estimated number of total malaria cases if mining between 2018 and 2023 mimicked historical patterns observed before 2018. The difference between estimated and reported cases refers to the excess malaria cases that occurred because of the increase in mining.
(DOCX)
