# Supplementary material for: Mining and malaria in the Brazilian Amazon and in the Yanomami indigenous land
Source: PLoS Negl Trop Dis. 2025 Nov 3;19(11):e0013677. doi: 10.1371/journal.pntd.0013677 (PMC12594400; doi:10.1371/journal.pntd.0013677)
Supplement: S1 Text — The green dots are the location of 301 villages that were mapped by the National Indigenous Foundation (Funai). The Indigenous Health Secretariat (SESAI) reports a total of 398 villages. Therefore, 97 villages do not have geographical coordinates. The numbers in the maps are the ID# of each subunit (polo base), as shown in the table below. Basemap: Natural Earth (Public Domain) https://www.naturalearthdata.com/about/terms-of-use/. Administrative boundaries (Brazil): geoBoundaries (CC BY 4.0) https://www.geoboundaries.org/countryDownloads.html. Indigenous lands (Brazil): FUNAI – Terras Indígenas (open government data; attribution required - see FUNAI page for terms) Main portal: https://www.gov.br/funai/pt-br/atuacao/terras-indigenas/geoprocessamento-e-mapas, Dataaccess/preview: https://geoserver.funai.gov.br/geoserver/web/wicket/bookmarkable/org.geoserver.web.demo.MapPreviewPage?filter=false. Fig B in S1 Text. Grid of weather data extracted from ERA5. Weather variables (temperature and rainfall) were aggregated to their associated subunit (polo base) by taking the weighted average of the area of the cells that overlapped each subunit. Basemap: Natural Earth (Public Domain) https://www.naturalearthdata.com/about/terms-of-use/. Fig C in S1 Text. Partial autocorrelation function (PAF) of climate variables by month. (A) Maximum temperature. (B) Total precipitation. (C) Oceanic Niño Index. Fig D in S1 Text. Diagnostic method used in reported malaria cases in the Brazilian Amazon and in the Yanomami indigenous land, 2014–2023. There was no missing information on diagnostic method except for the entire Amazon (1.8% in 2014, only 0.1% in 2023). Fig E in S1 Text. Type of detection (passive or active) of reported malaria cases in the Brazilian Amazon and in the Yanomami indigenous land, 2014–2023. There was no missing information on the type of detection. Table A in S1 Text. Variables included in the models. Two models were considered: (i) all malaria cases, and (ii) only loc [file pntd.0013677.s001.docx]

**Supplementary Information**

**Mining and malaria in the Brazilian Amazon and in the Yanomami indigenous land**

Marcia C. Castro^1^*, Nicholas J. Arisco^2^, Cesar Guerreiro Diniz^1,3,4^, Jamie Ponmattam^1^, Cassio Peterka^5^, Paulo Cesar Basta^6^, Marcelo Urbano Ferreira^7,8^

Contents

[Figure A. 2](#_Toc212220013)

[Figure B. 3](#_Toc212220014)

[Figure C. 4](#_Toc212220015)

[Figure D. 5](#_Toc212220016)

[Figure E. 6](#_Toc212220017)

[Table A. 7](#_Toc212220018)

[Table B. 8](#_Toc212220019)

[Table C. 9](#_Toc212220020)

[Table D. 10](#_Toc212220021)

[Table E. 11](#_Toc212220022)

[Table F. 12](#_Toc212220023)

[Table G. 13](#_Toc212220024)

[Table H. 14](#_Toc212220025)

[Table I. 15](#_Toc212220026)

[Table J. 16](#_Toc212220027)


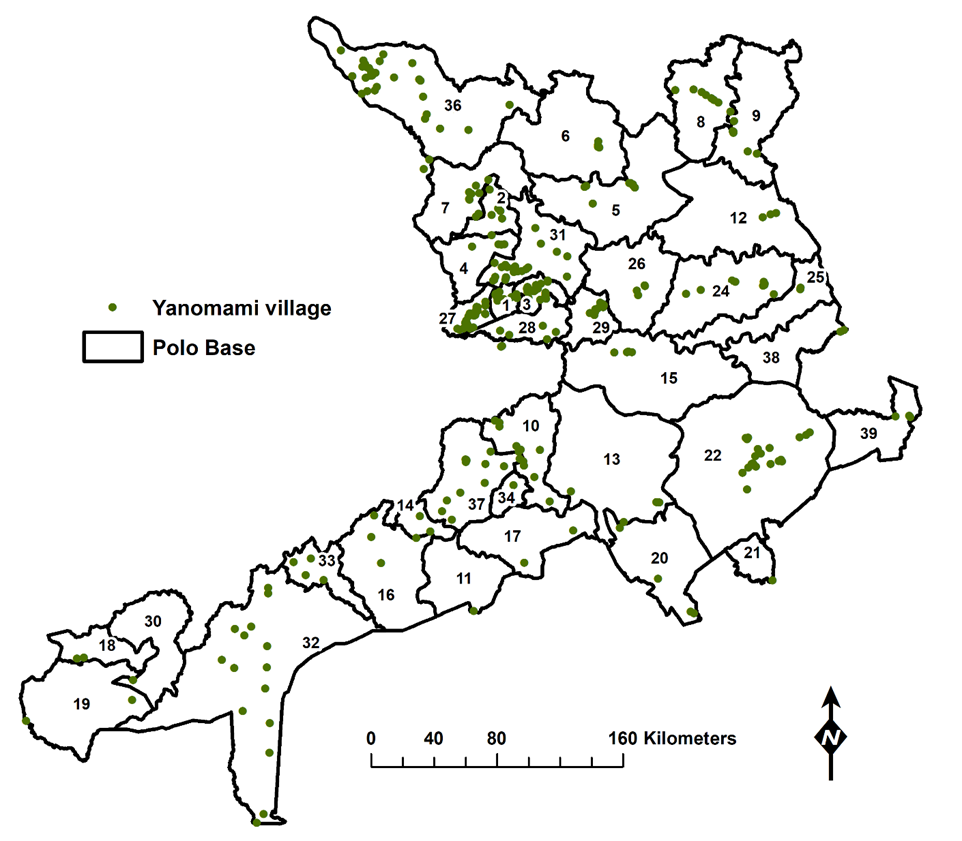


Figure A.

**Yanomami subunits and villages**. The green dots are the location of 301 villages that were mapped by the National Indigenous Foundation (Funai). The Indigenous Health Secretariat (SESAI) reports a total of 398 villages. Therefore, 97 villages do not have geographical coordinates. The numbers in the maps are the ID# of each subunit (*polo base*), as shown in the table below. Basemap: Natural Earth (Public Domain) [https://www.naturalearthdata.com/about/terms-of-use/](https://urldefense.proofpoint.com/v2/url?u=https-3A__www.naturalearthdata.com_about_terms-2Dof-2Duse_&d=DwMFaQ&c=WO-RGvefibhHBZq3fL85hQ&r=-Uxf0E9Knrr4v0XjfboOFVgzG3KT6BTM5BPUjGzPY0g&m=tchwwmTXGi6ciuMsBW7FpC1YzZSqmhMjBpFuk7BkATmalWCYg4fEKK1pvDFWs4Tk&s=t9hMmvWz6t6EvwmJDaYq_mi7XbGG4ucVUfIZoEDYANY&e=). Administrative boundaries (Brazil): geoBoundaries (CC BY 4.0) [https://www.geoboundaries.org/countryDownloads.html](https://urldefense.proofpoint.com/v2/url?u=https-3A__www.geoboundaries.org_countryDownloads.html&d=DwMFaQ&c=WO-RGvefibhHBZq3fL85hQ&r=-Uxf0E9Knrr4v0XjfboOFVgzG3KT6BTM5BPUjGzPY0g&m=tchwwmTXGi6ciuMsBW7FpC1YzZSqmhMjBpFuk7BkATmalWCYg4fEKK1pvDFWs4Tk&s=SEcmUHKt-eCeiGxf_FD-xQp7fQsV7OJXuzxBmPUvRms&e=). Indigenous lands (Brazil): FUNAI – Terras Indígenas (open government data; attribution required - see FUNAI page for terms) Main portal: [https://www.gov.br/funai/pt-br/atuacao/terras-indigenas/geoprocessamento-e-mapas](https://urldefense.proofpoint.com/v2/url?u=https-3A__www.gov.br_funai_pt-2Dbr_atuacao_terras-2Dindigenas_geoprocessamento-2De-2Dmapas&d=DwMFaQ&c=WO-RGvefibhHBZq3fL85hQ&r=-Uxf0E9Knrr4v0XjfboOFVgzG3KT6BTM5BPUjGzPY0g&m=tchwwmTXGi6ciuMsBW7FpC1YzZSqmhMjBpFuk7BkATmalWCYg4fEKK1pvDFWs4Tk&s=VZpzOE41pIhwQPUduccllDKZv8RBiNF0BVtMjQ1PxVk&e=), Data access/preview: [https://geoserver.funai.gov.br/geoserver/web/wicket/bookmarkable/org.geoserver.web.demo.MapPreviewPage?filter=false](https://urldefense.proofpoint.com/v2/url?u=https-3A__geoserver.funai.gov.br_geoserver_web_wicket_bookmarkable_org.geoserver.web.demo.MapPreviewPage-3Ffilter-3Dfalse&d=DwMFaQ&c=WO-RGvefibhHBZq3fL85hQ&r=-Uxf0E9Knrr4v0XjfboOFVgzG3KT6BTM5BPUjGzPY0g&m=tchwwmTXGi6ciuMsBW7FpC1YzZSqmhMjBpFuk7BkATmalWCYg4fEKK1pvDFWs4Tk&s=C1asdW9zl60xtgpnRjK-ISSzlr8ijPFLvRoF6OQr-nY&e=)

| **ID#** | **Subunit** | **ID#** | **Subunit** | **ID#** | **Subunit** | **ID#** | **Subunit** |
| --- | --- | --- | --- | --- | --- | --- | --- |
| 1 | Haxiu | 10 | Toototobi | 19 | Inambú | 29 | Maloca Paapiu |
| 2 | Aratha-u | 11 | Cachoeira do Araçá | 20 | Ajuricaba | 30 | Maia |
| 3 | Hakoma | 12 | Uraricoera | 21 | Baixo Catrimani | 31 | Surucucu |
| 4 | Waputha | 13 | Demini | 22 | Missão Catrimani | 32 | Marauiá |
| 5 | Palimiú | 14 | Alto Padauiri | 24 | Alto Mucajai | 33 | Marari |
| 6 | Waikás | 15 | Alto Catrimani | 25 | Baixo Mucajai | 34 | Novo-Demini |
| 7 | Parafuri | 16 | Médio Padauiri | 26 | Paapiu | 36 | Auaris |
| 8 | Sauba | 17 | Aracá | 27 | Xitei | 37 | Balawau |
| 9 | Ericó | 18 | Maturacá | 28 | Homoxi | 38 | Apiaú |
|  |  |  |  |  |  | 39 | Ajarani |


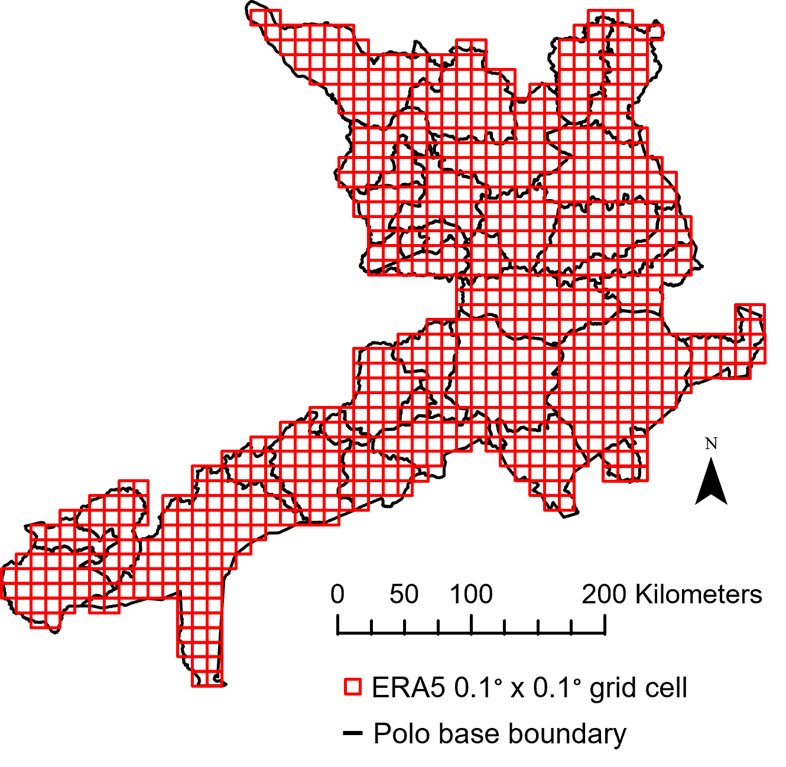


Figure B.

**Grid of weather data extracted from ERA5.** Weather variables (temperature and rainfall) were aggregated to their associated subunit (*polo base*) by taking the weighted average of the area of the cells that overlapped each subunit. Basemap: Natural Earth (Public Domain) [https://www.naturalearthdata.com/about/terms-of-use/](https://urldefense.proofpoint.com/v2/url?u=https-3A__www.naturalearthdata.com_about_terms-2Dof-2Duse_&d=DwMFaQ&c=WO-RGvefibhHBZq3fL85hQ&r=-Uxf0E9Knrr4v0XjfboOFVgzG3KT6BTM5BPUjGzPY0g&m=tchwwmTXGi6ciuMsBW7FpC1YzZSqmhMjBpFuk7BkATmalWCYg4fEKK1pvDFWs4Tk&s=t9hMmvWz6t6EvwmJDaYq_mi7XbGG4ucVUfIZoEDYANY&e=).


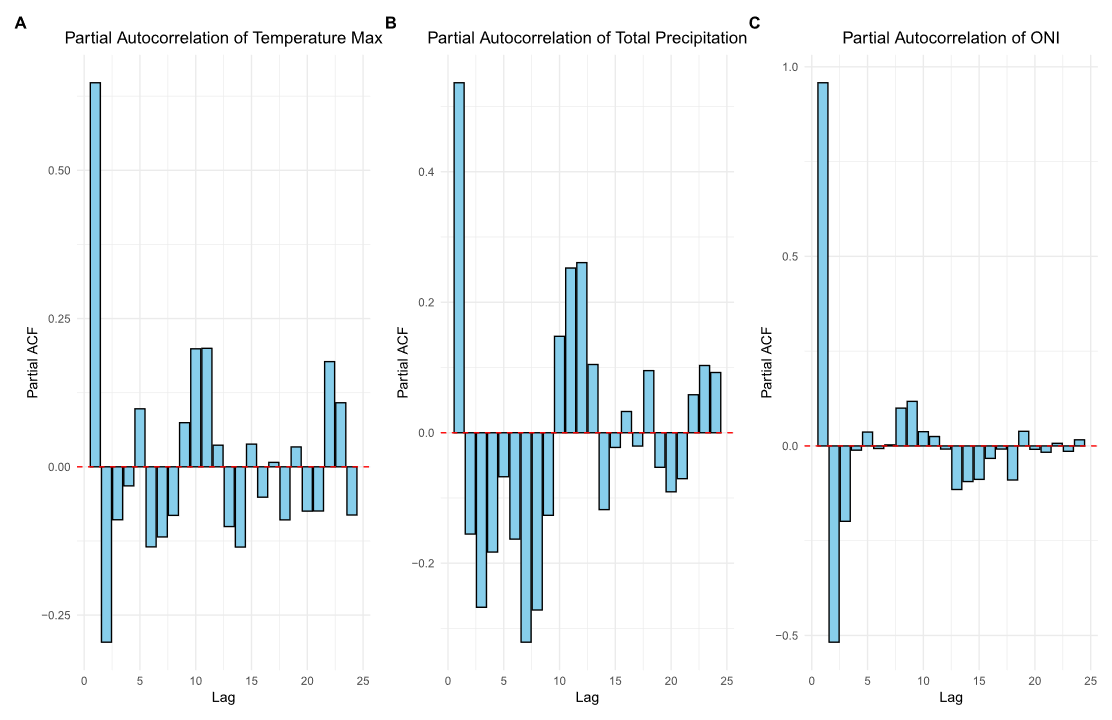


Figure C.

**Partial autocorrelation function (PAF) of climate variables by month**. **(A)** Maximum temperature. **(B)** Total precipitation. **(C)** Oceanic Niño Index.


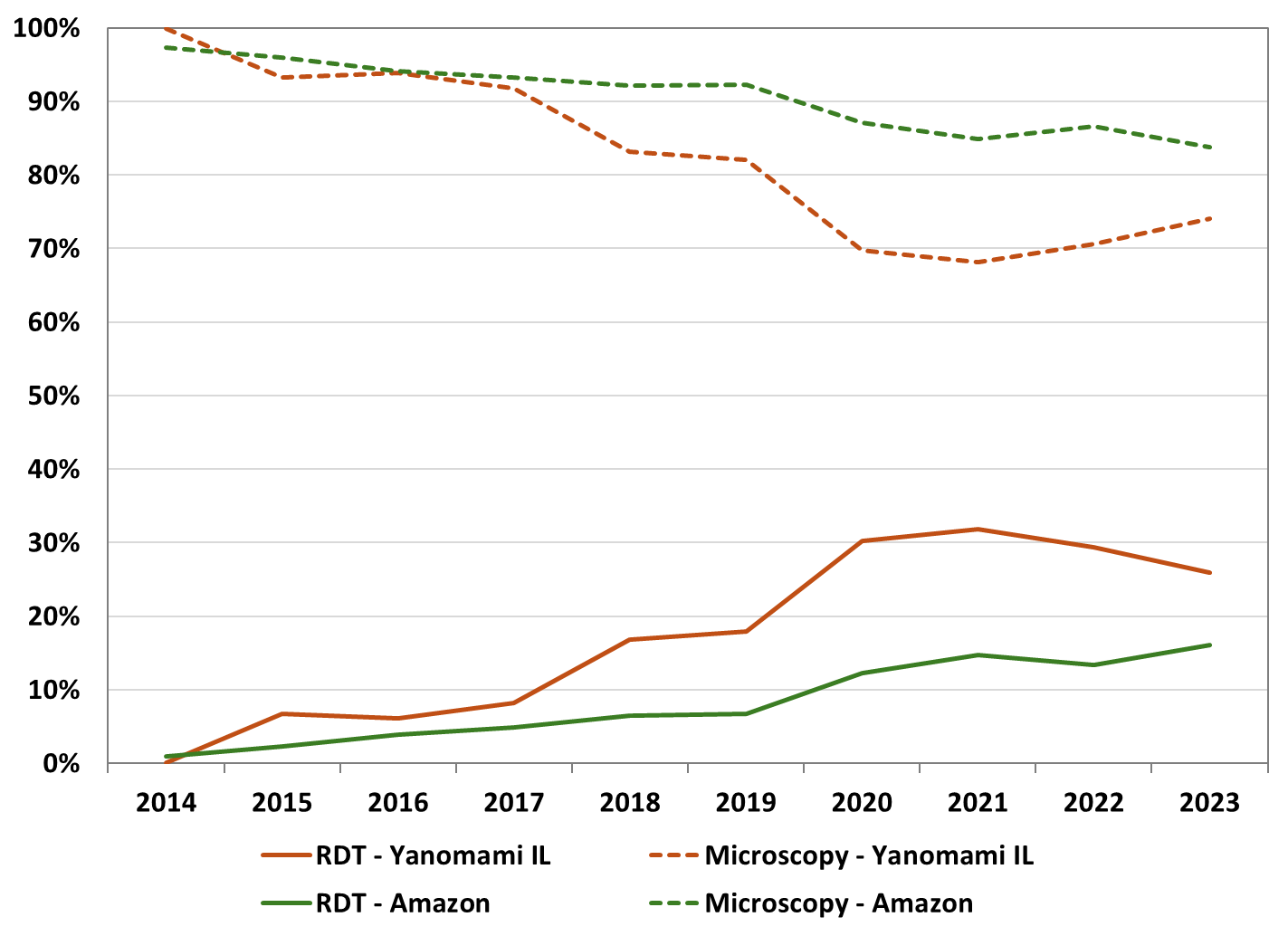


Figure D.

**Diagnostic** **method used in reported malaria cases in the Brazilian Amazon and in the Yanomami indigenous land, 2014-2023.** There was no missing information on diagnostic method except for the entire Amazon (1.8% in 2014, only 0.1% in 2023).


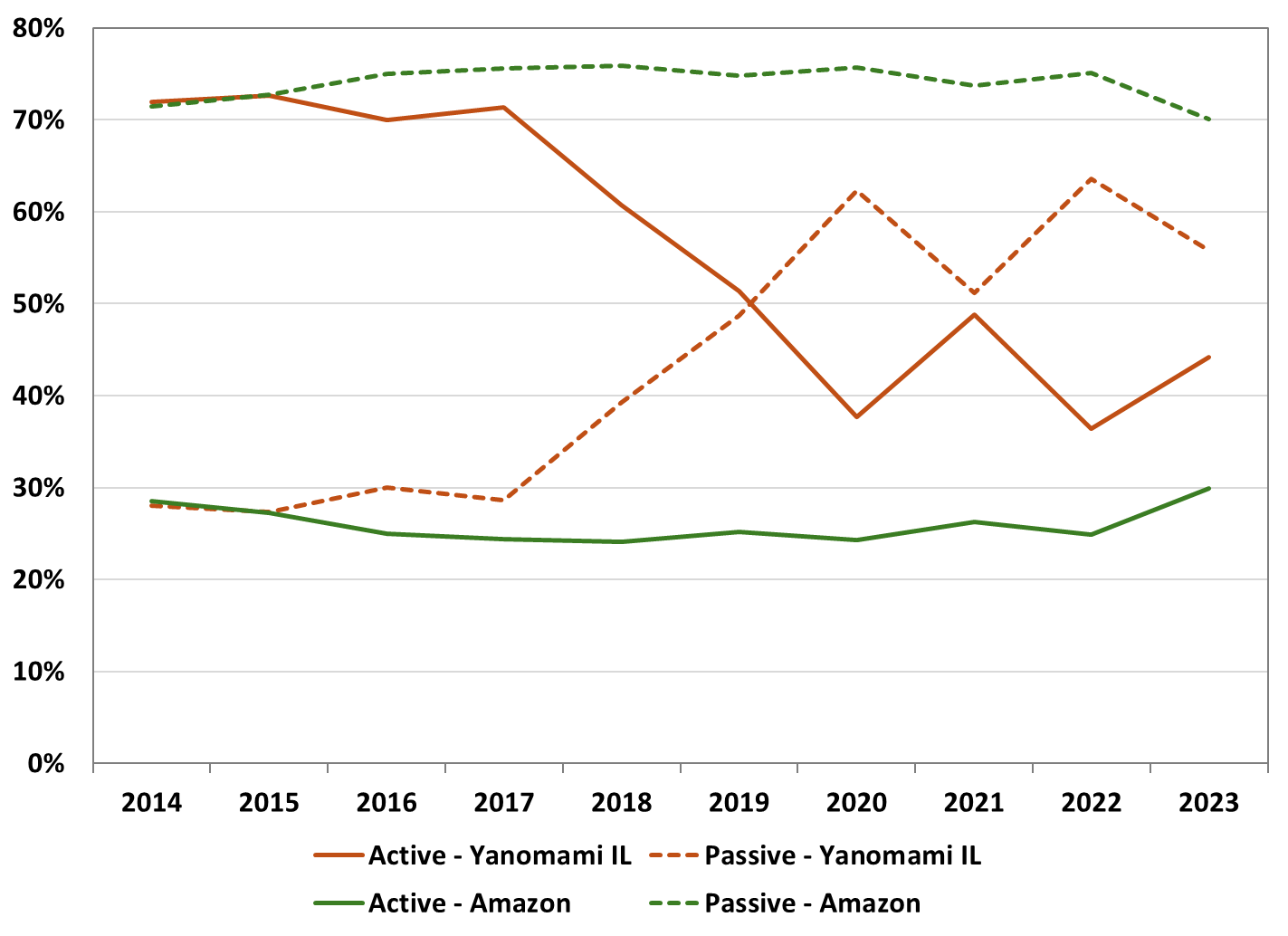


Figure E.

**Type of detection (passive or active) of reported malaria cases in the Brazilian Amazon and in the Yanomami indigenous land, 2014-2023.** There was no missing information on the type of detection.

Table A.

**Variables included in the models**. Two models were considered: (i) all malaria cases, and (ii) only locally acquired malaria cases.

| **Variable** | | **Type** | **Specification** | **Spatial Scale** | **Temporal Scale** | **Description** | **Source** | **Model** |
| --- | --- | --- | --- | --- | --- | --- | --- | --- |
| **Linear variables** | Malaria cases | Outcome | Count | Subunit | Monthly | Monthly malaria cases in which the infection and notification location were in the Yanomami territory | Sivep-Malaria | Both |
|  | Proportion of subunit mined | Predictor | Proportion | Subunit | Yearly | Annual cumulative area of subunit mined divided by the subunit area | MapBiomas | Both |
|  | Proportion of subunit deforested not related to mining | Predictor | Proportion | Subunit | Yearly | Annual cumulative area of subunit deforested but not related to mining, divided by the subunit area | MapBiomas | Both |
|  | Population of subunit | Predictor | Number | Subunit | Yearly | Estimated annual subunit population | SESAI | Both |
|  | Area | Predictor | Number | Subunit | Fixed | Area of the subunit | SESAI | Both |
| **Non-linear variables / random effects** | Maximum temperature | Predictor | 2^nd^ order autoregressive | Subunit | Monthly | Maximum monthly temperature at the centroid of each subunit | ERA5 | Both |
|  | Total precipitation | Predictor | 1^st^ order random walk | Subunit | Monthly | Total monthly precipitation at the centroid of each subunit | ERA5 | Both |
|  | Oceanic Niño Index | Predictor | 2^nd^ order random walk | Entire area | Monthly | ONI for the Yanomami territory | NOAA | Both |
|  | Year:Month | Predictor | Independent and identically distributed Gaussian random effect | Entire area | Yearly |  |  | Both |
|  | Month | Predictor | Fixed effect | Entire area | Monthly |  |  | Both |
|  | Year | Predictor | 1^st^ order autoregressive | Entire area | Yearly |  |  |  |
|  | Subunit | Predictor | Besag-York-Moille | Subunit | Fixed |  | SESAI | Both |
|  | Subunit | Predictor | Independent and identically distributed Gaussian random effect | Subunit | Fixed |  | SESAI | Both |

Table B.

**Prior specifications across all INLA models**. Prior specifications were chosen to be conservative in their information.

| **Variable** | **Hyperparameter Prior** | **Shape** | **Rate / Scale** |
| --- | --- | --- | --- |
| Subunit | PC Prec | 1 | 0.01 (Prob) |
| Subunit | Log-Gamma | 1 | 0.01 |
| Year | Log-Gamma | 5 | 0.01 |
| Month | Log-Gamma | 5 | 0.01 |
| Total monthly precipitation | Log-Gamma | 1 | 0.01 |
| Maximum monthly temperature | Log-Gamma | 1 | 0.01 |
| Oceanic Niño Index | Log-Gamma | 1 | 0.01 |

Table C.

**Area of *garimpo* in the Yanomami indigenous land by subunit, 2010-2023**. ID number corresponds to the map in Supplementary Figure 1.

| **Subunit** | **ID #** | ***Garimpo* area (Km^2^)** | | | | | | | | | | | | |  |
| --- | --- | --- | --- | --- | --- | --- | --- | --- | --- | --- | --- | --- | --- | --- | --- |
|  |  | **2010** | **2011** | **2012** | **2013** | **2014** | **2015** | **2016** | **2017** | **2018** | **2019** | **2020** | **2021** | **2022** | **2023** |
| Ajarani | 39 | 0 | 0 | 0 | 0 | 0 | 0 | 0 | 0 | 0 | 0 | 0 | 0 | 0 | 0 |
| Ajuricaba | 20 | 0 | 0 | 0 | 0 | 0 | 0 | 0 | 0 | 0 | 0 | 0 | 0 | 0 | 0 |
| Alto Catrimani | 15 | 0 | 0 | 0.009 | 0.023 | 0.016 | 0.012 | 0.007 | 0.008 | 0.008 | 0.140 | 0.421 | 1.010 | 1.605 | 1.605 |
| Alto Mucajai | 24 | 0 | 0 | 0 | 0 | 0 | 0 | 0 | 0 | 0 | 0 | 0 | 0.006 | 0.144 | 0.144 |
| Alto Padauiri | 14 | 0 | 0 | 0 | 0 | 0 | 0 | 0 | 0 | 0 | 0 | 0 | 0 | 0 | 0 |
| Apiaú | 38 | 0 | 0 | 0 | 0.004 | 0.009 | 0.005 | 0 | 0 | 0.028 | 0.068 | 0.221 | 0.459 | 0.672 | 0.672 |
| Aracá | 17 | 0 | 0 | 0 | 0 | 0 | 0 | 0 | 0 | 0 | 0 | 0 | 0 | 0 | 0 |
| Aratha-u | 2 | 0 | 0 | 0 | 0 | 0 | 0 | 0 | 0 | 0.007 | 0.07 | 0.066 | 0.066 | 0.29 | 0.29 |
| Auaris | 36 | 0 | 0 | 0 | 0 | 0 | 0 | 0 | 0.007 | 0.095 | 0.161 | 0.397 | 1.100 | 2.382 | 2.382 |
| Baixo Catrimani | 21 | 0 | 0 | 0 | 0 | 0 | 0 | 0 | 0 | 0 | 0 | 0 | 0 | 0 | 0 |
| Baixo Mucajai | 25 | 0 | 0 | 0 | 0 | 0 | 0 | 0 | 0 | 0 | 0 | 0 | 0 | 0 | 0 |
| Balawau | 37 | 0 | 0 | 0 | 0 | 0 | 0 | 0 | 0 | 0 | 0 | 0 | 0 | 0 | 0 |
| Cachoeira do Araçá | 11 | 0 | 0 | 0 | 0 | 0 | 0 | 0 | 0 | 0 | 0 | 0 | 0 | 0 | 0 |
| Demini | 13 | 0 | 0 | 0 | 0 | 0 | 0 | 0 | 0 | 0 | 0 | 0 | 0 | 0 | 0 |
| Ericó | 9 | 0 | 0 | 0.011 | 0.028 | 0.032 | 0.022 | 0.032 | 0.051 | 0.043 | 0.043 | 0.057 | 0.095 | 0.123 | 0.123 |
| Hakoma | 3 | 0 | 0 | 0 | 0 | 0 | 0 | 0 | 0.002 | 0.006 | 0.021 | 0.008 | 0.008 | 0.35 | 0.35 |
| Haxiu | 1 | 0 | 0 | 0 | 0 | 0 | 0 | 0 | 0 | 0 | 0 | 0 | 0 | 0 | 0 |
| Homoxi | 28 | 0.01 | 0.005 | 0.006 | 0.011 | 0.009 | 0 | 0.013 | 0.013 | 0.012 | 0.028 | 0.186 | 1.367 | 6.099 | 6.099 |
| Inambú | 19 | 0 | 0 | 0 | 0 | 0 | 0 | 0 | 0 | 0 | 0 | 0 | 0 | 0 | 0 |
| Maia | 30 | 0 | 0 | 0 | 0 | 0 | 0 | 0 | 0 | 0 | 0 | 0 | 0 | 0 | 0 |
| Maloca Paapiu | 29 | 0 | 0 | 0 | 0.011 | 0.011 | 0 | 0 | 0.031 | 0.031 | 0.031 | 0.029 | 0.171 | 0.425 | 0.425 |
| Marari | 33 | 0 | 0 | 0 | 0 | 0 | 0 | 0 | 0 | 0 | 0 | 0 | 0 | 0 | 0 |
| Marauiá | 32 | 0 | 0 | 0 | 0 | 0 | 0 | 0 | 0 | 0 | 0 | 0 | 0 | 0 | 0 |
| Maturacá | 18 | 0 | 0 | 0 | 0 | 0 | 0 | 0 | 0 | 0 | 0 | 0 | 0 | 0 | 0 |
| Médio Padauiri | 16 | 0 | 0 | 0 | 0 | 0 | 0 | 0 | 0 | 0 | 0 | 0 | 0 | 0 | 0 |
| Missão Catrimani | 22 | 0 | 0 | 0 | 0 | 0 | 0 | 0 | 0 | 0 | 0 | 0 | 0 | 0 | 0 |
| Novo-Demini | 34 | 0 | 0 | 0 | 0 | 0 | 0 | 0 | 0 | 0 | 0 | 0 | 0 | 0 | 0 |
| Paapiu | 26 | 0.021 | 0.002 | 0.016 | 0.019 | 0.013 | 0.004 | 0 | 0.021 | 0.500 | 1.880 | 2.749 | 4.953 | 8.733 | 8.733 |
| Palimiú | 5 | 0 | 0 | 0 | 0 | 0 | 0 | 0 | 0 | 0 | 0 | 0 | 0 | 0 | 0 |
| Parafuri | 7 | 0 | 0 | 0 | 0 | 0 | 0 | 0 | 0 | 0 | 0 | 0.058 | 0.290 | 0.47 | 0.47 |
| Sauba | 8 | 0 | 0 | 0 | 0 | 0 | 0 | 0 | 0 | 0 | 0 | 0 | 0 | 0 | 0 |
| Surucucu | 31 | 0 | 0 | 0.007 | 0.003 | 0.027 | 0.029 | 0.01 | 0.005 | 0 | 0.001 | 0.007 | 0.017 | 0.533 | 0.533 |
| Toototobi | 10 | 0 | 0 | 0 | 0 | 0 | 0 | 0 | 0 | 0 | 0 | 0 | 0 | 0 | 0 |
| Uraricoera | 12 | 0 | 0 | 0 | 0 | 0 | 0 | 0 | 0 | 0 | 0 | 0 | 0 | 0 | 0 |
| Waikás | 6 | 0.003 | 0.002 | 0 | 0 | 0.002 | 0.008 | 0.201 | 1.075 | 3.425 | 5.193 | 5.977 | 8.702 | 12.745 | 12.745 |
| Waputha | 4 | 0 | 0 | 0 | 0 | 0 | 0 | 0 | 0 | 0 | 0 | 0 | 0 | 0 | 0 |
| Xitei | 27 | 0 | 0.001 | 0.001 | 0 | 0 | 0 | 0 | 0 | 0 | 0 | 0 | 0.09 | 2.116 | 2.116 |
| **Total** |  | **0.034** | **0.010** | **0.050** | **0.099** | **0.119** | **0.080** | **0.263** | **1.213** | **4.155** | **7.636** | **10.180** | **18.330** | **36.687** | **36.687** |

Table D.

**Reported malaria cases in the Yanomami indigenous land by subunit of infection, 2010-2023**. ID number corresponds to the map in Supplementary Figure 1.

| **Subunit** | **ID #** | **Malaria cases** | | | | | | | | | | | | | |  |
| --- | --- | --- | --- | --- | --- | --- | --- | --- | --- | --- | --- | --- | --- | --- | --- | --- |
|  |  | **2010** | **2011** | **2012** | **2013** | **2014** | **2015** | **2016** | **2017** | **2018** | **2019** | **2020** | **2021** | **2022** | **2023** | |
| Ajarani | 39 | 105 | 84 | 144 | 152 | 119 | 29 | 2 | 47 | 31 | 8 | 7 | 22 | 14 | 30 | |
| Ajuricaba | 20 | 2 | 18 | 9 | 0 | 0 | 3 | 12 | 82 | 89 | 43 | 79 | 254 | 378 | 322 | |
| Alto Catrimani | 15 | 9 | 2 | 0 | 0 | 0 | 48 | 178 | 5 | 0 | 180 | 241 | 439 | 311 | 308 | |
| Alto Mucajai | 24 | 497 | 222 | 14 | 1 | 74 | 474 | 296 | 57 | 485 | 554 | 976 | 821 | 431 | 891 | |
| Alto Padauiri | 14 | 76 | 66 | 9 | 40 | 11 | 29 | 34 | 127 | 166 | 171 | 107 | 252 | 197 | 233 | |
| Apiaú | 38 | 61 | 48 | 11 | 1 | 16 | 73 | 17 | 6 | 138 | 39 | 127 | 350 | 221 | 230 | |
| Aracá | 17 | 64 | 3 | 16 | 26 | 15 | 3 | 29 | 235 | 88 | 125 | 22 | 72 | 30 | 111 | |
| Aratha-u | 2 | 59 | 34 | 0 | 21 | 156 | 60 | 423 | 142 | 156 | 859 | 2,212 | 1,953 | 1,832 | 1,659 | |
| Auaris | 36 | 795 | 464 | 69 | 77 | 10 | 32 | 27 | 56 | 294 | 710 | 2,427 | 2,279 | 1,284 | 8,263 | |
| Baixo Catrimani | 21 | 23 | 137 | 80 | 19 | 0 | 10 | 1 | 90 | 236 | 126 | 64 | 370 | 303 | 560 | |
| Baixo Mucajai | 25 | 330 | 114 | 31 | 19 | 169 | 237 | 75 | 7 | 230 | 50 | 285 | 269 | 56 | 181 | |
| Balawau | 37 | 101 | 284 | 76 | 9 | 1 | 21 | 426 | 201 | 604 | 978 | 768 | 1,373 | 1,319 | 1,078 | |
| Cachoeira do Araçá | 11 | 22 | 2 | 13 | 45 | 58 | 8 | 58 | 171 | 137 | 282 | 174 | 180 | 101 | 69 | |
| Demini | 13 | 7 | 7 | 0 | 0 | 0 | 0 | 2 | 34 | 30 | 23 | 4 | 63 | 53 | 96 | |
| Ericó | 9 | 143 | 172 | 39 | 215 | 103 | 241 | 349 | 5 | 125 | 377 | 501 | 583 | 85 | 290 | |
| Hakoma | 3 | 1 | 1 | 0 | 0 | 0 | 1 | 41 | 7 | 0 | 24 | 6 | 56 | 213 | 833 | |
| Haxiu | 1 | 1 | 0 | 1 | 0 | 2 | 4 | 0 | 0 | 6 | 1 | 28 | 2 | 4 | 216 | |
| Homoxi | 28 | 2 | 0 | 0 | 0 | 0 | 0 | 0 | 0 | 0 | 5 | 25 | 17 | 7 | 487 | |
| Inambú | 19 | 13 | 8 | 4 | 0 | 2 | 2 | 9 | 28 | 194 | 289 | 110 | 49 | 12 | 88 | |
| Maia | 30 | 239 | 8 | 0 | 3 | 2 | 7 | 69 | 100 | 110 | 116 | 49 | 10 | 31 | 18 | |
| Maloca Paapiu | 29 | 17 | 33 | 0 | 0 | 0 | 72 | 9 | 1 | 38 | 218 | 559 | 922 | 691 | 1,612 | |
| Marari | 33 | 2,033 | 800 | 674 | 993 | 1,038 | 693 | 343 | 1,349 | 1,030 | 2,151 | 1,728 | 1,964 | 424 | 1,293 | |
| Marauiá | 32 | 460 | 742 | 194 | 147 | 316 | 1,495 | 2,686 | 3,111 | 2,156 | 3,423 | 2,874 | 2,139 | 2,054 | 1,941 | |
| Maturacá | 18 | 108 | 19 | 7 | 1 | 1 | 11 | 13 | 36 | 78 | 151 | 106 | 31 | 37 | 69 | |
| Médio Padauiri | 16 | 381 | 280 | 617 | 664 | 520 | 290 | 490 | 532 | 694 | 924 | 487 | 774 | 753 | 564 | |
| Missão Catrimani | 22 | 642 | 414 | 37 | 75 | 189 | 213 | 324 | 807 | 933 | 261 | 682 | 890 | 1,339 | 1,229 | |
| Novo-Demini | 34 | 82 | 36 | 37 | 2 | 7 | 12 | 26 | 153 | 403 | 573 | 695 | 1,265 | 538 | 1,077 | |
| Paapiu | 26 | 204 | 4 | 0 | 0 | 0 | 101 | 0 | 0 | 151 | 576 | 687 | 475 | 175 | 126 | |
| Palimiú | 5 | 149 | 268 | 19 | 0 | 43 | 141 | 48 | 0 | 293 | 657 | 1,736 | 409 | 1,112 | 2,653 | |
| Parafuri | 7 | 216 | 141 | 12 | 0 | 3 | 283 | 157 | 392 | 171 | 749 | 1,012 | 747 | 703 | 1,244 | |
| Sauba | 8 | 46 | 59 | 6 | 21 | 60 | 53 | 269 | 9 | 169 | 246 | 299 | 575 | 116 | 129 | |
| Surucucu | 31 | 25 | 14 | 0 | 1 | 3 | 74 | 6 | 2 | 2 | 58 | 237 | 491 | 583 | 1,667 | |
| Toototobi | 10 | 134 | 21 | 103 | 7 | 2 | 8 | 75 | 346 | 583 | 1,128 | 983 | 893 | 532 | 1,196 | |
| Uraricoera | 12 | 80 | 95 | 14 | 0 | 26 | 30 | 128 | 11 | 126 | 681 | 1,066 | 533 | 319 | 451 | |
| Waikás | 6 | 9 | 12 | 0 | 0 | 2 | 1 | 13 | 11 | 122 | 1,653 | 2,319 | 4,127 | 9,350 | 3,982 | |
| Waputha | 4 | 0 | 2 | 0 | 0 | 0 | 3 | 0 | 0 | 5 | 26 | 29 | 18 | 15 | 205 | |
| Xitei | 27 | 13 | 26 | 0 | 0 | 0 | 5 | 1 | 12 | 61 | 102 | 16 | 15 | 27 | 105 | |
| **Total** | | **7,149** | **4,640** | **2,236** | **2,539** | **2,948** | **4,767** | **6,636** | **8,172** | **10,134** | **18,537** | **23,727** | **25,682** | **25,650** | **35,506** | |

Table E.

**Malaria Annual Parasite Index (API) per 100 people in the Yanomami indigenous land by subunit of infection, 2010-2023**. ID number corresponds to the map in Supplementary Figure 1.

| **Subunit** | **ID #** | **Annual Parasite Index (API = cases by 100 people)** | | | | | | | | | | | | |  |
| --- | --- | --- | --- | --- | --- | --- | --- | --- | --- | --- | --- | --- | --- | --- | --- |
|  |  | **2010** | **2011** | **2012** | **2013** | **2014** | **2015** | **2016** | **2017** | **2018** | **2019** | **2020** | **2021** | **2022** | **2023** |
| Ajarani | 39 | 171.9 | 136.2 | 255.4 | 258.6 | 201.7 | 48.3 | 3.5 | 85.2 | 46.3 | 10.2 | 17.1 | 58.3 | 36.1 | 48.6 |
| Ajuricaba | 20 | 0.9 | 6.0 | 2.5 | 0.0 | 0.0 | 0.7 | 3.7 | 20.1 | 19.9 | 9.0 | 16.5 | 51.7 | 75.6 | 56.9 |
| Alto Catrimani | 15 | 4.7 | 1.0 | 0.0 | 0.0 | 0.0 | 20.6 | 74.3 | 2.0 | 0.0 | 62.7 | 67.4 | 130.3 | 64.2 | 57.8 |
| Alto Mucajai | 24 | 106.5 | 46.2 | 2.8 | 0.0 | 12.6 | 83.7 | 49.8 | 9.5 | 80.6 | 87.0 | 148.7 | 124.7 | 61.2 | 121.5 |
| Alto Padauiri | 14 | 48.1 | 39.4 | 4.7 | 20.1 | 6.0 | 14.7 | 17.1 | 63.3 | 80.7 | 80.3 | 48.9 | 114.6 | 85.8 | 102.2 |
| Apiaú | 38 | 70.1 | 52.2 | 11.5 | 1.0 | 15.2 | 69.5 | 12.5 | 5.1 | 90.1 | 24.8 | 53.6 | 170.1 | 103.8 | 97.7 |
| Aracá | 17 | 22.5 | 1.0 | 4.6 | 7.8 | 4.4 | 0.6 | 6.5 | 105.0 | 38.3 | 54.5 | 8.4 | 30.0 | 10.2 | 39.3 |
| Aratha-u | 2 | 11.0 | 6.1 | 0.0 | 3.7 | 26.9 | 9.8 | 66.5 | 22.5 | 22.5 | 124.5 | 274.6 | 165.9 | 174.8 | 204.7 |
| Auaris | 36 | 27.1 | 14.8 | 2.1 | 2.3 | 0.2 | 0.8 | 0.6 | 1.2 | 7.1 | 16.5 | 59.1 | 54.8 | 29.2 | 171.1 |
| Baixo Catrimani | 21 | 24.7 | 140.2 | 78.0 | 17.3 | 0.0 | 8.3 | 0.0 | 57.4 | 142.3 | 72.4 | 31.2 | 186.0 | 137.7 | 224.2 |
| Baixo Mucajai | 25 | 153.6 | 50.0 | 11.8 | 7.2 | 61.7 | 83.9 | 26.2 | 2.3 | 71.4 | 15.7 | 96.6 | 89.6 | 16.1 | 51.8 |
| Balawau | 37 | 15.9 | 44.0 | 11.4 | 1.2 | 0.1 | 2.8 | 58.1 | 26.3 | 76.6 | 120.3 | 95.8 | 166.5 | 156.2 | 123.8 |
| Cachoeira do Araçá | 11 | 25.3 | 0.0 | 13.3 | 34.1 | 58.4 | 8.5 | 44.2 | 151.5 | 120.0 | 240.0 | 159.8 | 143.4 | 81.1 | 48.6 |
| Demini | 13 | 4.8 | 4.0 | 0.0 | 0.0 | 0.0 | 0.0 | 0.5 | 14.9 | 12.0 | 7.3 | 1.4 | 26.8 | 22.0 | 38.6 |
| Ericó | 9 | 63.3 | 71.8 | 16.0 | 84.7 | 40.2 | 89.3 | 114.5 | 1.3 | 35.8 | 105.2 | 131.3 | 153.7 | 20.5 | 66.8 |
| Hakoma | 3 | 0.0 | 0.2 | 0.0 | 0.0 | 0.0 | 0.2 | 6.8 | 0.8 | 0.0 | 3.6 | 0.8 | 7.4 | 29.1 | 118.0 |
| Haxiu | 1 | 0.0 | 0.0 | 0.2 | 0.0 | 0.1 | 0.4 | 0.0 | 0.0 | 0.4 | 0.1 | 2.7 | 0.2 | 0.2 | 19.4 |
| Homoxi | 28 | 0.2 | 0.0 | 0.0 | 0.0 | 0.0 | 0.0 | 0.0 | 0.0 | 0.0 | 0.7 | 8.2 | 4.5 | 0.4 | 182.5 |
| Inambú | 19 | 3.1 | 1.5 | 0.9 | 0.0 | 0.3 | 0.3 | 1.9 | 5.0 | 37.0 | 53.8 | 19.4 | 8.6 | 1.9 | 11.4 |
| Maia | 30 | 45.5 | 1.5 | 0.0 | 0.0 | 0.2 | 1.2 | 10.6 | 16.4 | 17.0 | 16.3 | 6.4 | 1.5 | 4.5 | 2.6 |
| Maloca Paapiu | 29 | 3.6 | 8.9 | 0.0 | 0.0 | 0.0 | 17.9 | 2.1 | 0.0 | 8.4 | 46.7 | 114.5 | 188.9 | 135.9 | 296.3 |
| Marari | 33 | 265.7 | 120.0 | 98.0 | 140.4 | 142.1 | 88.3 | 43.5 | 164.0 | 119.3 | 240.5 | 189.2 | 212.2 | 56.0 | 158.1 |
| Marauiá | 32 | 23.5 | 36.8 | 9.3 | 6.9 | 14.4 | 62.4 | 107.1 | 122.5 | 81.4 | 122.2 | 101.0 | 72.6 | 65.3 | 59.4 |
| Maturacá | 18 | 7.0 | 1.0 | 0.1 | 0.0 | 0.0 | 0.1 | 0.6 | 1.5 | 3.1 | 6.3 | 3.8 | 1.1 | 1.2 | 2.3 |
| Médio Padauiri | 16 | 83.8 | 59.6 | 127.0 | 133.3 | 100.0 | 53.2 | 86.4 | 87.7 | 107.7 | 139.4 | 63.8 | 105.2 | 74.1 | 57.1 |
| Missão Catrimani | 22 | 88.0 | 56.1 | 4.7 | 9.4 | 23.2 | 23.1 | 36.4 | 89.6 | 102.0 | 26.3 | 72.2 | 93.6 | 133.6 | 110.2 |
| Novo-Demini | 34 | 20.1 | 8.9 | 8.8 | 0.5 | 1.6 | 2.4 | 4.6 | 21.8 | 53.8 | 74.5 | 66.0 | 114.5 | 45.3 | 85.9 |
| Paapiu | 26 | 92.3 | 1.8 | 0.0 | 0.0 | 0.0 | 40.9 | 0.0 | 0.0 | 49.3 | 179.2 | 206.7 | 143.7 | 51.4 | 85.9 |
| Palimiú | 5 | 23.6 | 42.1 | 2.7 | 0.0 | 6.0 | 17.7 | 5.7 | 0.0 | 33.7 | 71.1 | 154.1 | 35.3 | 90.6 | 205.0 |
| Parafuri | 7 | 64.9 | 40.4 | 3.4 | 0.0 | 0.8 | 66.1 | 34.7 | 85.5 | 36.3 | 154.3 | 192.5 | 126.5 | 129.5 | 239.9 |
| Sauba | 8 | 15.2 | 17.0 | 1.6 | 6.3 | 17.8 | 14.7 | 76.6 | 2.7 | 50.3 | 72.9 | 105.7 | 201.4 | 36.2 | 36.9 |
| Surucucu | 31 | 1.2 | 0.7 | 0.0 | 0.1 | 0.1 | 3.6 | 0.2 | 0.0 | 0.0 | 1.5 | 8.9 | 18.3 | 20.1 | 55.5 |
| Toototobi | 10 | 26.1 | 4.0 | 18.6 | 1.2 | 0.3 | 1.3 | 11.2 | 54.4 | 93.4 | 173.2 | 214.9 | 190.9 | 107.4 | 229.8 |
| Uraricoera | 12 | 64.7 | 69.7 | 10.2 | 0.0 | 19.0 | 15.5 | 83.0 | 0.7 | 18.9 | 168.7 | 695.0 | 256.2 | 110.3 | 253.6 |
| Waikás | 6 | 4.7 | 8.3 | 0.0 | 0.0 | 0.0 | 0.6 | 4.5 | 5.4 | 17.2 | 78.2 | 100.5 | 277.2 | 187.6 | 123.5 |
| Waputha | 4 | 0.0 | 0.2 | 0.0 | 0.0 | 0.0 | 0.3 | 0.0 | 0.0 | 0.4 | 2.0 | 2.9 | 2.3 | 1.8 | 22.3 |
| Xitei | 27 | 0.6 | 1.7 | 0.0 | 0.0 | 0.0 | 0.2 | 0.1 | 0.5 | 3.0 | 5.3 | 0.6 | 0.3 | 0.6 | 4.1 |
| **Total** | | **1,584.1** | **1,093.2** | **699.5** | **736.2** | **753.0** | **851.9** | **993.8** | **1,226.2** | **1,676.3** | **2,667.2** | **3,540.1** | **3,728.8** | **2,357.4** | **3,813.2** |

Table F.

**Model results for different time periods, outcomes, and configuration of mining data.**

| **Model** | **Posterior Median** | **SD** | **95% Credible Interval** |  |
| --- | --- | --- | --- | --- |
|  |  |  |  |  |
|  |  |  |  |  |
| Observed mining |  |  |  |  |
| 2010-2023 |  |  |  |  |
| Total malaria cases | 1.24 | 1.03 | 1.17, 1.32 |  |
| Locally acquired malaria cases | 1.19 | 1.03 | 1.11, 1.27 |  |
| 2010-2017 |  |  |  |  |
| Total malaria cases | 0.98 | 1.05 | 0.89, 1.08 |  |
| Locally acquired malaria cases | 0.99 | 1.05 | 0.90, 1.11 |  |
| 2018-2023 |  |  |  |  |
| Total malaria cases | 1.22 | 1.04 | 1.13, 1.33 |  |
| Locally acquired malaria cases | 1.18 | 1.05 | 1.07, 1.29 |  |
| 1-km buffered area around observed mining |  |  |  |  |
| 2010-2023 |  |  |  |  |
| Total malaria cases | 1.23 | 1.04 | 1.15, 1.32 |  |
| Locally acquired malaria cases | 1.22 | 1.04 | 1.13, 1.31 |  |
| 2010-2017 |  |  |  |  |
| Total malaria cases | 1.13 | 1.10 | 0.97, 1.39 |  |
| Locally acquired malaria cases | 1.20 | 1.10 | 0.99, 1.44 |  |
| 2018-2023 |  |  |  |  |
| Total malaria cases | 1.15 | 1.05 | 1.05, 1.26 |  |
| Locally acquired malaria cases | 1.14 | 1.05 | 1.03, 1.27 |  |
| 5-km buffered area around observed mining |  |  |  |  |
| 2010-2023 |  |  |  |  |
| Total malaria cases | 1.34 | 1.04 | 1.25, 1.44 |  |
| Locally acquired malaria cases | 1.30 | 1.04 | 1.21, 1.39 |  |
| 2010-2017 |  |  |  |  |
| Total malaria cases | 1.22 | 1.11 | 0.99, 1.51 |  |
| Locally acquired malaria cases | 1.27 | 1.12 | 1.01, 1.59 |  |
| 2018-2023 |  |  |  |  |
| Total malaria cases | 1.18 | 1.06 | 1.06, 1.31 |  |
| Locally acquired malaria cases | 1.20 | 1.06 | 1.08, 1.35 |  |

Table G.

**Additional fixed effects included in model (relevant to model that used observed mining as the outcome).**

| **Model** | **Posterior Median** | **SD** | **95% Credible Interval** |  |
| --- | --- | --- | --- | --- |
|  |  |  |  |  |
|  |  |  |  |  |
| Proportion of subunit deforested |  |  |  |  |
| (excluding deforestation from mining) |  |  |  |  |
| 2010-2023 |  |  |  |  |
| Total malaria cases | 0.84 | 1.03 | 0.79, 0.88 |  |
| Locally acquired malaria cases | 0.83 | 1.03 | 0.78, 0.87 |  |
| 2010-2017 |  |  |  |  |
| Total malaria cases | 0.81 | 1.04 | 0.75, 0.87 |  |
| Locally acquired malaria cases | 0.81 | 1.04 | 0.74, 0.87 |  |
| 2018-2023 |  |  |  |  |
| Total malaria cases | 0.90 | 1.05 | 0.81, 0.99 |  |
| Locally acquired malaria cases | 0.90 | 1.06 | 0.80, 1.01 |  |
| Population of subunit |  |  |  |  |
| 2010-2023 |  |  |  |  |
| Total malaria cases | 1.36 | 1.13 | 1.07, 1.74 |  |
| Locally acquired malaria cases | 1.44 | 1.13 | 1.12, 1.84 |  |
| 2010-2017 |  |  |  |  |
| Total malaria cases | 0.78 | 1.26 | 0.49, 1.22 |  |
| Locally acquired malaria cases | 0.74 | 1.29 | 0.45, 1.2 |  |
| 2018-2023 |  |  |  |  |
| Total malaria cases | 1.56 | 1.26 | 1.00, 2.46 |  |
| Locally acquired malaria cases | 1.73 | 1.26 | 1.11, 2.74 |  |
| Area of subunit |  |  |  |  |
| 2010-2023 |  |  |  |  |
| Total malaria cases | 1.46 | 1.26 | 0.94, 2.29 |  |
| Locally acquired malaria cases | 1.38 | 1.28 | 0.86, 2.24 |  |
| 2010-2017 |  |  |  |  |
| Total malaria cases | 1.83 | 1.31 | 1.08, 3.15 |  |
| Locally acquired malaria cases | 1.90 | 1.34 | 1.08, 3.41 |  |
| 2018-2023 |  |  |  |  |
| Total malaria cases | 1.50 | 1.28 | 0.92, 2.43 |  |
| Locally acquired malaria cases | 1.37 | 1.28 | 0.84, 2.24 |  |

Table H.

**Sensitivity analysis of the temporal specification of the model**. For DIC and WAIC, lower numbers indicate a better fit; for logCPO, higher numbers indicate a better fit. The model specification used for both locally acquired malaria cases and total malaria cases is indicated by an asterisk (*).

| **Month Specification** | **Year Specification** | **Outcome: Locally acquired malaria cases** | | | **Outcome: Total malaria cases** | | |
| --- | --- | --- | --- | --- | --- | --- | --- |
|  |  | **DIC** | **WAIC** | **LogCPO** | **DIC** | **WAIC** | **LogCPO** |
| Month in Year, Random Effect | Auto Regressive Order 1 | 39,657.15 | 39,685.46 | -19,847.61 | 41,169.84 | 41,196.95 | -20,603.35 |
| Month in Year, Random Effect | Random Walk Order 1 | 39,657.29 | 39,685.07 | -19,848.13 | 41,171.79 | 41,199.95 | -20,604.87 |
| Month in Year, Random Effect | Random Walk Order 2 | 39,647.64 | 39,674.35 | -19,841.84 | 41,162.09 | 41,189.05 | -20,598.82 |
| Month in Year, Random Effect | Fixed effect | 39,646.90 | 39,674.44 | -19,842.11 | 41,162.81 | 41,190.14 | -20,599.49 |
| Month in Year, Random Effect & Month Fixed Effect (*) | Auto Regressive Order 1 | 39,646.26 | 39,675.19 | -19,842.58 | 41,162.59 | 41,190.62 | -20,599.68 |
| Month in Year, Random Effect & Month Fixed Effect | Random Walk Order 1 | 39,646.20 | 39,674.94 | -19,842.47 | 41,163.80 | 41,190.97 | -20,600.01 |
| Random Effect | Random Walk Order 1 | 39,695.29 | 39,718.20 | -19,860.89 | 41,206.64 | 41,228.27 | -20,615.75 |
| Random Walk Order 1 | Random Walk Order 1 | 39,697.90 | 39,719.51 | -19,861.47 | 41,207.77 | 41,229.32 | -20,616.22 |
| Random Walk Order 1 | Fixed effect | 39,696.77 | 39,719.34 | -19,861.43 | 41,209.11 | 41,230.21 | -20,616.70 |
| sin(2 * pi * (month / 12)) + cos(2 * pi * (month / 12)) | Fixed effect | 39,696.80 | 39,717.83 | -19,860.48 | 41,210.69 | 41,231.47 | -20,617.22 |
| Random Walk Order 2 | Fixed effect | 39,698.21 | 39,719.64 | -19,861.51 | 41,211.35 | 41,232.13 | -20,617.63 |
| Year:Month Interaction, Random Walk Order 1 | N/A | 39,681.18 | 39,713.93 | -19,865.46 | 41,198.53 | 41,229.34 | -20,622.87 |

Table I.

**Distributional assumption sensitivity analysis**. For DIC and WAIC, lower numbers indicate a better fit; for logCPO, higher numbers indicate a better fit. The model specification used for both locally acquired malaria cases and total malaria cases is indicated by an asterisk (*).

| **Distributional Assumption** | **Outcome: Locally acquired malaria cases** | | | **Outcome: Total malaria cases** | | | |
| --- | --- | --- | --- | --- | --- | --- | --- |
|  | **DIC** | **WAIC** | **LogCPO** | **DIC** | **WAIC** | **LogCPO** |  |
| Zero-inflated Poisson | 267,857.93 | 2,748,326.79 | -128,457.11 | 287,433.77 | 1,863,193.77 | -158,993.28 |  |
| Zero-inflated Negative Binomial (*) | 42,003.99 | 42,003.97 | -21,001.99 | 43,682.74 | 43,682.73 | -21,841.37 |  |
| Negative Binomial | 42,020.78 | 42,020.43 | -21,010.21 | 43,638.28 | 43,638.01 | -21,819.01 |  |
| Poisson | 408,449.85 | 1,968,685.35 | -193,264.94 | 430,374.44 | 2,479,487.80 | -225,533.57 |  |

Table J.

Estimates for simulated scenarios. Scenario A = estimated number of locally acquired malaria cases in 2023. The difference between estimated and reported cases refers to the likely underreporting of locally acquired cases. Scenario B = estimated number of total malaria cases if mining between 2018 and 2023 mimicked historical patterns observed before 2018. The difference between estimated and reported cases refers to the excess malaria cases that occurred because of the increase in mining.

| **Subunit** | **ID #** | **Scenario A** | | | **Scenario B** | | |
| --- | --- | --- | --- | --- | --- | --- | --- |
|  |  | **Estimated malaria cases in 2022** | **Reported malaria cases in 2022** | **Estimated -**  **Reported** | **Estimated malaria cases, 2018 to 2023** | **Reported malaria cases 2018 to 2023** | **Estimated**  **-**  **Reported** |
| Ajarani | 39 | 295 | 13 | 282 | 1,029 | 112 | 917 |
| Ajuricaba | 20 | 151 | 347 | -196 | 316 | 1,165 | -849 |
| Alto Catrimani | 15 | 316 | 181 | 135 | 646 | 1,479 | -833 |
| Alto Mucajai | 24 | 918 | 407 | 511 | 1,639 | 4,158 | -2,519 |
| Alto Padauiri | 14 | 235 | 187 | 48 | 448 | 1,126 | -678 |
| Apiaú | 38 | 197 | 217 | -20 | 322 | 1,105 | -783 |
| Aracá | 17 | 168 | 25 | 143 | 566 | 448 | 118 |
| Aratha-u | 2 | 1,155 | 1,260 | -105 | 1,084 | 8,671 | -7,587 |
| Auaris | 36 | 2,535 | 1,255 | 1,280 | 784 | 15,257 | -14,473 |
| Baixo Catrimani | 21 | 279 | 252 | 27 | 562 | 1,659 | -1,097 |
| Baixo Mucajai | 25 | 427 | 55 | 372 | 1,060 | 1,071 | -11 |
| Balawau | 37 | 1,008 | 1,307 | -299 | 1,326 | 6,120 | -4,794 |
| Cachoeira do Araçá | 11 | 226 | 90 | 136 | 451 | 943 | -492 |
| Demini | 13 | 62 | 53 | 9 | 241 | 269 | -28 |
| Ericó | 9 | 553 | 80 | 473 | 1,184 | 1,961 | -777 |
| Hakoma | 3 | 307 | 195 | 112 | 242 | 1,132 | -890 |
| Haxiu | 1 | 93 | 2 | 91 | 140 | 257 | -117 |
| Homoxi | 28 | 338 | 1 | 337 | 390 | 541 | -151 |
| Inambú | 19 | 171 | 12 | 159 | 115 | 742 | -627 |
| Maia | 30 | 197 | 31 | 166 | 522 | 334 | 188 |
| Maloca Paapiu | 29 | 719 | 689 | 30 | 340 | 4,040 | -3,700 |
| Marari | 33 | 2,541 | 400 | 2,141 | 6,565 | 8,590 | -2,025 |
| Marauiá | 32 | 4,551 | 1,845 | 2,706 | 5,025 | 14,587 | -9,562 |
| Maturacá | 18 | 165 | 28 | 137 | 164 | 472 | -308 |
| Médio Padauiri | 16 | 1,869 | 674 | 1,195 | 3,388 | 4,196 | -808 |
| Missão Catrimani | 22 | 1,010 | 1,291 | -281 | 1,928 | 5,334 | -3,406 |
| Novo-Demini | 34 | 699 | 491 | 208 | 365 | 4,551 | -4,186 |
| Paapiu | 26 | 1,601 | 161 | 1,440 | 956 | 2,190 | -1,234 |
| Palimiú | 5 | 1,102 | 1,102 | 0 | 810 | 6,860 | -6,050 |
| Parafuri | 7 | 769 | 658 | 111 | 1,181 | 4,626 | -3,445 |
| Sauba | 8 | 302 | 109 | 193 | 631 | 1,534 | -903 |
| Surucucu | 31 | 564 | 550 | 14 | 176 | 3,038 | -2,862 |
| Toototobi | 10 | 782 | 510 | 272 | 912 | 5,315 | -4,403 |
| Uraricoera | 12 | 405 | 150 | 255 | 459 | 3,176 | -2,717 |
| Waikás | 6 | 396 | 377 | 19 | 112 | 21,553 | -21,441 |
| Waputha | 4 | 68 | 15 | 53 | 167 | 298 | -131 |
| Xitei | 27 | 437 | 13 | 424 | 121 | 326 | -205 |
| **Total** | | **27,611** | **15,033** | **12,578** | **36,366** | **139,236** | **-102,870** |
